# Pauciflorins A–E, Unexpected Chromone–Monoterpene-Derived Meroterpenoids from *Centrapalus pauciflorus*

**DOI:** 10.1021/acs.jnatprod.2c01132

**Published:** 2023-03-18

**Authors:** Gordana Krstić, Muhammad Bello Saidu, Petra Bombicz, Sourav De, Hazhmat Ali, István Zupkó, Róbert Berkecz, Umar Shehu Gallah, Dóra Rédei, Judit Hohmann

**Affiliations:** † Department of Pharmacognosy, University of Szeged, Eötvös u. 6, 6720 Szeged, Hungary; ‡ Faculty of Chemistry, University of Belgrade, Studentski trg 12-16, 11158 Belgrade, Serbia; § Centre for Structural Science, Research Centre for Natural Sciences, Magyar Tudósok körútja 2, 1117 Budapest, Hungary; ⊥ Institute of Pharmacodynamics and Biopharmacy, University of Szeged, Eötvös u. 6, 6720 Szeged, Hungary; ∥ Institute of Pharmaceutical Analysis, University of Szeged, Somogyi u. 4, 6720 Szeged, Hungary; ▽ Bioresource Department, National Research Institute for Chemical Technology (NARICT), Zaria 1052, Nigeria; ○ ELKH-USZ Biologically Active Natural Products Research Group, University of Szeged, Eötvös u. 6, H-6720 Szeged, Hungary

## Abstract

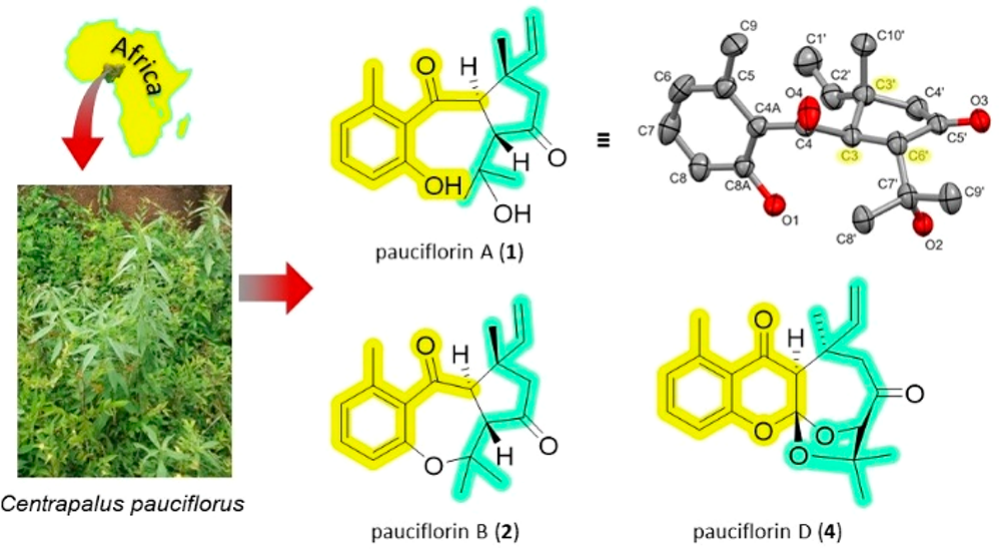

Five unusual meroterpenoids based on new carbon skeletons, pauciflorins A–E (**1**–**5**), were isolated by multistep chromatographic separations of a methanol extract of the aerial parts of *Centrapalus pauciflorus*. Compounds **1**–**3** are derived by the connection of a 2-nor-chromone and a monoterpene unit, whereas **4** and **5** are dihydrochromone–monoterpene adducts with a rarely occurring orthoester functionality. The structures were solved using 1D and 2D NMR, HRESIMS, and single-crystal X-ray diffraction. Pauciflorins A–E were evaluated for antiproliferative activity against human gynecological cancer cell lines, but were inactive (IC_50_ < 10 μM) in each case.

Meroterpenoids are natural products derived from hybrid terpenoid and polyketide or non-polyketide biosynthesis. These compounds consist of a ring system involving a mono-, sesqui-, or diterpenoid moiety and a phloroglucinol, syncarpic acid, phthalide, benzofuran, phenylfuran, chromane/chromone, coumarin, quinone, flavone, or alkaloid component.^1^ Exceptionally diverse and complex structures are derived from connections of the structural fragments of different biosynthetic origins. Monoterpenoid-coupled chromones are rare compounds in plants that usually occur together with structurally related monoterpenoid coumarins in the genera of the family Asteraceae.^2−4^ Species of the Nassauvieae, Mutisieae, and Vernonieae tribes from the Asteraceae family were found to synthesize monoterpenoid-type meroterpenes. *Nassauvia aculeata*,^5^*Triptilion benauentei*, and *T. spinosum*(6) from Nassauvieae, *Gerbera piloselloides*,^2^*G. delavayi*,^3^ and *Mutisia friesiana*(7) from Mutisieae, and *Bothriocline ripensis*(8) from the Vernonieae tribe were reported to accumulate both coumarin- and chromone-based meroterpenoids, while only coumarin-monoterpene-derived meroterpenoids were isolated previously from *Gutenbergia*,^8^*Ethulia*,^9^ and *Vernonia*(10) species of the Vernonieae tribe. Chromone-based meroterpenoids exhibit cytotoxic, antiproliferative, and anti-inflammatory activities.^2^

*Centrapalus pauciflorus* (Willd.) H.Rob. (Asteraceae family, Vernonieae tribe) was investigated as part of an ongoing effort to discover new bioactive metabolites from African plant species. This species is native to tropical African regions and is found predominantly in the Western and Eastern countries of the continent.^11^*C. pauciflorus* has been used in traditional medicine to treat chest and stomach pain.^12^ In a preliminary experiment, fractions obtained from the chloroform-soluble extract of *C. pauciflorus* were assayed against the human breast (MCF-7 and MDA-MB-231), cervical (HeLa), and ovarian (A2780) cancer cell lines for antiproliferative activity. As presented in Figure S1 in the Supporting Information, fraction 3 eluted with 60% MeOH from the polyamide column exhibited the most potent activity; so therefore this fraction was selected for isolation of the chemical constituents. The present paper reports the isolation and structural determination of five chromone–monoterpene-type meroterpenoids (**1**–**5**) (Figure 1) from the leaves of *C. pauciflorus*.

**Figure 1:**
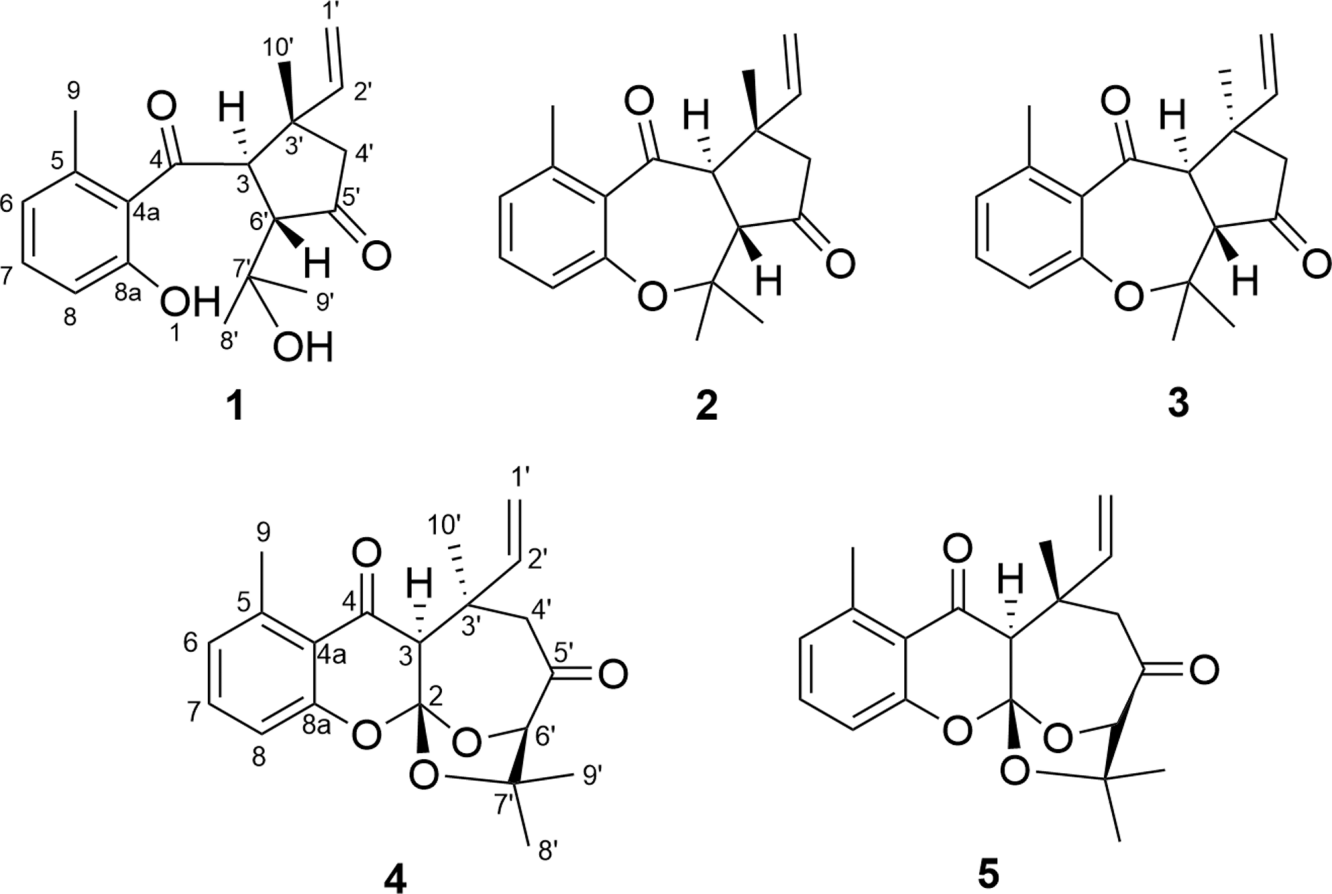
Structures of compounds **1**–**5**.

## Results and Discussion

**Figure 2:**
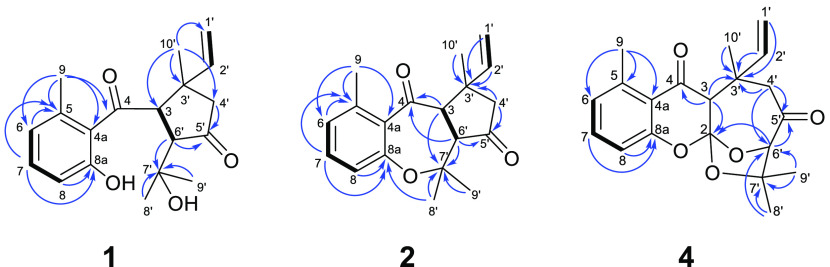
Key ^1^H–^1^H COSY (**−**) and HMBC (blue →) correlations of compounds **1**, **2**, and **4**.

**Figure 3:**
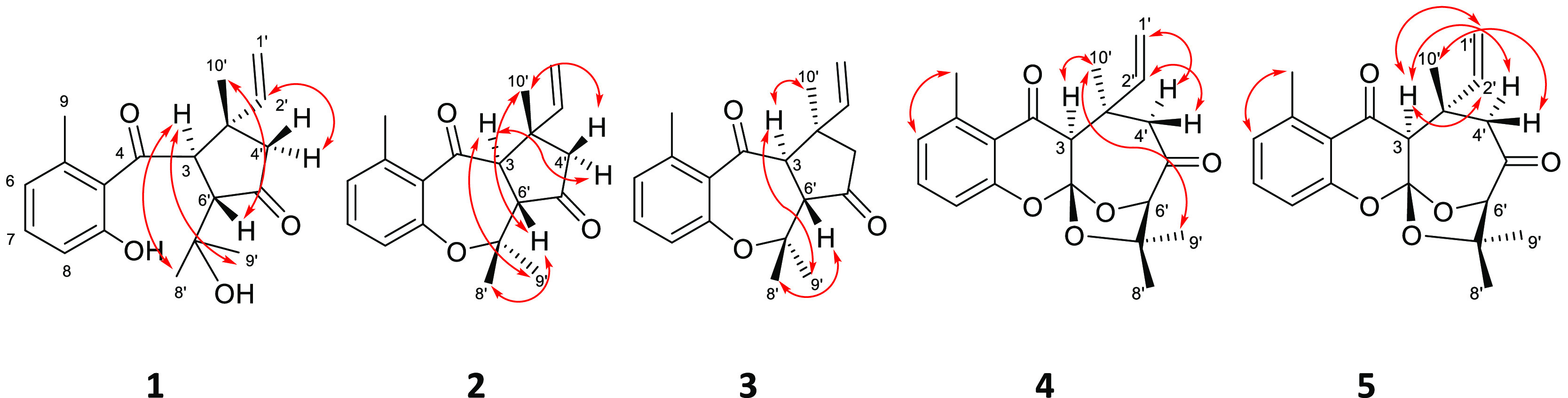
Key NOESY correlations (H↔H) of compounds **1**–**5**.

**Figure 4:**
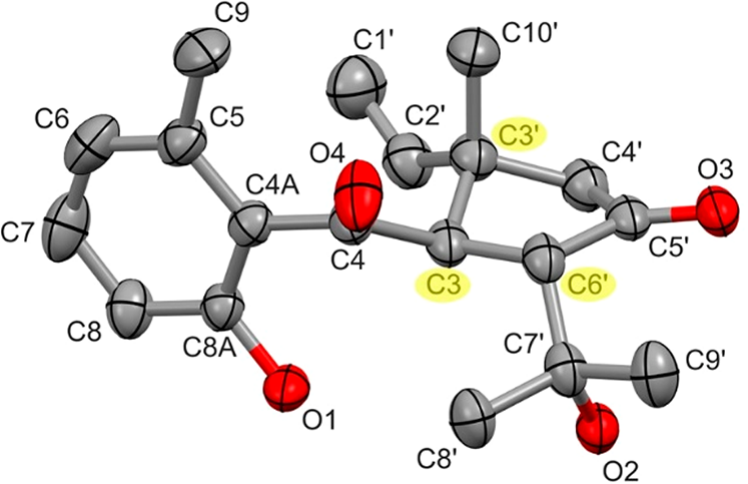
ORTEP presentation of compound **1**. The displacement ellipsoids are drawn at the 50% probability level. The chiral centers are highlighted by yellow, C3 (*R*), C3′ (*S*), and C6′ (*S*).

Pauciflorin A (**1**) was isolated as a colorless oily material with an optical rotation of [α]_D_ −157.8 (*c* 0.1, CHCl_3_). The molecular formula of **1** was determined as C_19_H_24_O_4_, based on the positive-ion HRESIMS peak at *m*/*z* 339.1567 [M + Na]^+^ (calcd for C_19_H_24_O_4_Na^+^ 339.1567). The ^1^H and ^13^C NMR JMOD spectra revealed characteristic resonances of four methyl, two methylene, six methine, and seven quaternary carbon-containing groups (Tables 1 and 2). The aromatic ^1^H NMR resonances at δ_H_ 6.71 d (8.0 Hz), 7.20 t (8.0 Hz), and 6.78 d (8.0 Hz) indicated a 1,2,3-trisubstituted aromatic ring, which was substituted with a methyl (δ_H_ 2.39 s, δ_C_ 21.5), a hydroxy, and a carbonyl group (δ_C_ 205.9). The position of the methyl group at C-5 was shown by the HMBC correlations between H-9 (δ_H_ 2.39) and C-5 (δ_C_ 137.5), C-4a (δ_C_ 127.3), and C-6 (δ_C_ 123.4) (Figure 2). A monoterpene moiety was elucidated as 2-(1-hydroxyisopropyl)-4-methyl-4-vinyl-1-cyclopentanone and was coupled with the aromatic unit at position C-3. The monoterpene fragment was displayed by ^1^H–^1^H COSY correlations of H-3 [δ_H_ 4.15 d (11.3 Hz)] with H-6′ [δ_H_ 3.43 d (11.3 Hz)], and H_2_-1′ [δ_H_ 4.89 d (17.2 Hz) and 4.78 d (10.6 Hz)] with H-2′ [δ_H_ 5.65 dd (17.2, 10.6 Hz)] and by the HMBC correlations of H-10 (δ_H_ 1.04 s) with C-3 (δ_C_ 59.2), C-2′ (δ_C_ 143.1), C-3′ (δ_C_ 43.9), and C-4′ (δ_C_ 55.7); and H-3, H-8′ (δ_H_ 1.56 s), and H-9′ (δ_H_ 1.23 s) with C-6′ (δ_C_ 59.7) and C-7′ (δ_C_ 73.1) (Figure 2). The long-range heteronuclear correlations of H-3 (δ_H_ 4.15 d) and H-6′ (δ_H_ 3.43 d) with C-4 confirmed the connectivity of the C-4 carbonyl group (δ_C_ 205.9) with the aromatic ring and monoterpene unit. The stereochemistry of compound **1** was investigated by NOESY spectroscopy. NOE enhancements were observed between H-3/H-8′, H-3/H-9′, H-2′/H-4′α, and H-6′/H-10′, indicating α*-*oriented H-3 and a vinyl group and the β-orientation of H-10′ and H-6′ (Figure 3). The carbon skeleton of **1** has a 2-norchromone monoterpene origin, which has not been described previously. The structure and configuration of compound **1** were confirmed using X-ray crystallography (Figure 4).

**Table 1:** ^1^H NMR Spectroscopic Data of Compounds **1**–**3** [500 MHz, CDCl_3_, δ ppm (*J* = Hz)]

position	**1**	**2**	**3**
3	4.15, d (11.3)	3.21, d (11.6)	3.10, d (11.6)
6	6.71, d (8.0)	7.05, d (8.0)	7.04, d (8.0)
7	7.20, t (8.0)	7.30, t (8.0)	7.28, t (8.0)
8	6.78, d (8.0)	6.83, d (8.0)	6.80, d (8.0)
9	2.39, s	2.43, s	2.39, s
1′a	4.89, d (17.2)	5.12, d (17.5)	4.98, d (17.5)
1′b	4.78, d (10.6)	5.17, d (11.0)	5.04, d (11.0)
2′	5.65, dd (17.2, 10.6)	6.51, dd (17.5, 11.0)	5.90, dd (17.5, 11.0)
4′α	2.42, d (17.1)	2.51, d (18.0)	2.27, d (18.0)
4′β	2.23, d (17.1)	2.27, d (18.0)	2.60, d (18.0)
6′	3.43, d (11.3)	2.73, d (11.6)	2.72, d (11.6)
8′	1.56, s	1.51, s	1.50, s
9′	1.23, s	1.34, s	1.34, s
10′	1.04, s	1.12, s	1.73, s

**Table 2:** ^13^C NMR Spectroscopic Data of Compounds **1**–**5** (125 MHz, CDCl_3_, δ ppm)

no.	**1**	**2**	**3**	**4**	**5**
2				123.8, C	123.7, C
3	59.2, CH	58.4, CH	59.1, CH	63.3, CH	62.7, CH
4	205.9, C	204.5, C	205.9, C	191.7, C	191.1, C
4a	127.3, C	132.2, C	132.7, C	119.0, C	119.1, C
5	137.5, C	140.0, C	139.6, C	142.4, C	142.5, C
6	123.4, CH	128.5, CH	128.4, CH	126.1, CH	126.3, CH
7	132.6, CH	132.2, CH	132.0, CH	134.8, CH	134.7, CH
8	116.4, CH	123.5, CH	123.3, CH	115.8, CH	115.9, CH
8a	156.4, C	154.4, C	154.0, C	158.3, C	158.4, C
9	21.5, CH_3_	20.6, CH_3_	20.1, CH_3_	23.0, CH_3_	23.1, CH_3_
1′	113.4, CH_2_	112.1, CH_2_	115.0, CH_2_	114.3, CH_2_	111.3, CH_2_
2′	143.1, CH	145.7, CH	140.8, C	142.3, CH	148.6, CH
3′	43.9, C	43.5, C	44.0, C	41.0, C	40.6, C
4′	55.7, CH_2_	54.4, CH_2_	53.8, CH_2_	57.2, CH_2_	56.2, CH_2_
5′	214.4, C	213.4, C	213.5, C	211.0, C	211.8, C
6′	59.7, CH	56.3, CH	56.8, CH	87.0, CH	86.7, CH
7′	73.1, C	80.5, C	80.6, C	85.1, C	84.9, C
8′	30.4, CH_3_	26.1, CH_3_	26.1, CH_3_	27.8, CH_3_	27.6, CH_3_
9′	23.8, CH_3_	22.4, CH_3_	22.3, CH_3_	21.1, CH_3_	21.2, CH_3_
10′	20.4, CH_3_	20.7, CH_3_	27.3, CH_3_	32.2, CH_3_	20.0, CH_3_

Pauciflorin B (**2**) was obtained as a white amorphous powder with an optical rotation of [α]^27^_D_ −270.8 (*c* 0.1, CHCl_3_). The molecular formula of **2** was assigned as C_19_H_22_O_3_ from the protonated molecular ion at *m*/*z* 299.1641 [M + H]^+^ (calcd for C_19_H_23_O_3_ 299.1642) observed in the positive-ion HRESIMS. The ^1^H NMR and ^13^C NMR JMOD spectroscopic data of **2** revealed the presence of a 1,2,3-trisubstituted aromatic ring [δ_H_ 6.71 d (8.0 Hz), 7.20 t (8.0 Hz), and 6.78 d (8.0 Hz); δ_C_ 123.5, 128.5, 2 × 132.2, 140.0, and 154.4], four tertiary methyl groups (δ_H_ 1.12, 1.34, 1.51, and 2.43 s; δ_C_ 20.6, 20.7, 22.4, and 26.1), and a vinyl group [δ_H_ 5.12 d (17.5 Hz), 5.17 d (11.0 Hz), and 6.51 dd (17.5, 11.0 Hz); δ_C_ 112.1 and 145.7] (Tables 1 and 2). Two carbonyl functionalities were evident from the carbon resonances at δ_C_ 204.5 and 213.4. The structural units, quaternary carbons (δ_C_ 43.7 and 80.5), methines [δ_H_ 3.21 d (11.6 Hz) 2.73 d (11.6 Hz); δ_C_ 58.4 and 56.3], and a methylene [δ_H_ 2.27 d (18.0 Hz) and 2.51 d (18.0 Hz); δ_C_ 54.4] were connected by HMBC correlations, yielding a tricyclic compound that may originate from **1** by the loss of H_2_O. The structure of **2** was verified by the following HMBC correlations: H-3 and H-6′ with C-4; H-7, H-8, and H_3_-8′ with C-8a; H-3, H-6′, H_3_-8, and H_3_-9 with C-7′; H-6′ and H_2_-4 with C-5′; H-3, H-1′, H-2′, H-10′, and H_2_-4′ with C-3′ (Figure 2). After determining the planar structure, the relative configuration of compound **2** was analyzed by NOESY spectroscopy. Starting from the β-oriented H-6′, H_3_-10′, H_3_-9′, and H-4′b were assigned as β, and H-3, H_3_-8′, and H-4a as α according to the NOESY cross-peaks detected between H-6′/H_3_-8′, H-6′/H_3_-10′, H_3_-10′/H-4′β, H-3/H_3_-9′, and H-3/H-4α (Figure 3). The absolute configuration at C-3, C-3′, and C-6′ was inferred as *R*, *S*, and *S*, respectively, after considering the structural similarity of compound **2** with pauciflorin A (**1**).

Pauciflorin C (**3**) was obtained as a white amorphous powder with an optical rotation of [α]^27^_D_ +67.6 (*c* 0.05, CHCl_3_). It gave a molecular formula of C_19_H_22_O_3_ based on the positive-ion HRESIMS peak at *m*/*z* 299.1646 [M + H]^+^ (calcd for C_19_H_23_O_3_ 299.1642). The 1D (^1^H NMR and JMOD) and 2D NMR (^1^H–^1^H COSY, HSQC, and HMBC) data revealed compound **3** to be a diastereomer of **2** (Tables 1 and 2). The different ^1^H and ^13^C NMR chemical shifts of H-3, H-1′, H_2_-2′, H_3_-10′, C-4, C-1′, C-2′, and C-10′ suggested that **2** and **3** differ in the configuration of C-3′. This was corroborated by the key NOESY correlations between H-3/H_3_-10′, H-3/H_3_-9′, and H-6′/H_3_-8′ (Figure 3). The absolute configuration of compound **3** was proposed as 3*R*, 3′*R*, and 6′*S*.

Pauciflorin D (**4**) was obtained as a colorless oily material with an optical rotation of [α]^26^_D_ +6.9 (*c* 0.05, CHCl_3_). Its molecular formula was assigned as C_20_H_22_O_5_ according to the positive-ion HRESIMS spectrum, which gave a molecular ion peak at *m*/*z* 343.1539 [M + H]^+^ (calcd for C_20_H_23_O_5_ 345.1540), indicating 10 degrees of unsaturation (DUs). The NMR data showed the occurrence of a chromone–monoterpene hybrid motif in compound **4** (Tables 2 and 3). The 5-methylchromone part of **4** was found to be the same as the (C-3–C-9) part of compounds **1**–**3**, with **4** containing also an additional quaternary carbon (C-2, δ_C_ 123.8). The C-1′–C-5′ monoterpene part of **4** was also similar to those of compounds **1**–**3**; a difference was noted in the C-2, C-6′–C-9′ portions of the molecules. This structural part of compound **4** was elucidated from the presence of two additional oxygen atoms related to **2** and **3** (as shown by HRESIMS), HMBC correlations between H-3/C-2, H-6′/C-2, H-4′b/C-6′, H_3_-8′/C-6′, H_3_-9′/C-6′, H_3_-8′/C-7′, and H_3_-9′/C-7′, and O-bearing quaternary carbons at δ_C_ 158.3 (C-8a), 123.8 (C-2), 87.0 (C-6′), and 85.1 (C-7′) (Figure 2). With regard to the two carbonyl groups (δ_C_ 191.7 and 211.0), one vinyl group [δ_H_ 5.17 d (18.0 Hz), 5.11 d (11.0 Hz), and 6.26 dd (18.0 and 11.0 Hz); δ_C_ 114.3 and 142.3], and an aromatic ring [δ_H_ 6.88 d (7.8 Hz), 7.35 t (7.8 Hz), and 6.90 d (7.8 Hz); δ_C_ 119.0, 142.4, 126.1, 134.8, 115.8, and 158.3], a tetracyclic ring system was required to satisfy the remaining four DUs. As shown in Figure 2, an analysis of the ^1^H–^1^H COSY, HSQC, and HMBC spectra revealed the planar structure of **4** (Figure 1). A NOESY experiment was performed to assign the relative configuration of **4**. NOESY cross-peaks were observed between H-3/H_3_-10′ and H_3_-10′/H-4′α, indicating an α-oriented 10′-methyl group and H-3 and a β-oriented vinyl group. A *gem*-dimethyl-substituted-C-7′–O bridge was identified from the NOESY correlations between H-4′β/H-2′ and H-2′/H-9′ (Figure 3). The other O-bridge between C-2–C-6′ was proposed as being in the α-orientation. Consequently, pauciflorin D (**4**) was elucidated with an unprecedented tetracyclic heterocyclic ring system, as shown in Figure 1.

**Table 3:** ^1^H NMR Spectroscopic Data of Compounds **4** and **5** [500 MHz, CDCl_3_, δ ppm (*J* = Hz)]

no.	**4**	**5**
3	3.35, s	3.50, s
7	6.88, d (7.8)	6.87, d (7.8)
8	7.35, t (7.8)	7.34, t (7.8)
9	6.90, d (7.8)	6.90, d (7.8)
11	2.64, s	2.63, s
1a′	5.11, d (11.0)	5.09, d (17.4)
1b′	5.17, d (18.0)	5.05, d (10.7)
2′	6.26, dd (18.0, 11.0)	6.20, dd (17.4, 10.7)
4′α	2.50, d (13.0)	3.39, d (12.5)
4′β	3.36, d (13.0)	2.18, d (12.5)
6′	4.19, s	4.22, s
8′	1.54, s	1.56, s
9′	1.29, s	1.37, s
10′	1.60, s	1.37, s

Pauciflorin E (**5**) was isolated as a colorless oily material with optical rotation of [α]^27^_D_ +21.9 (*c* 0.1, CHCl_3_). The molecular formula was determined as C_20_H_22_O_5_ based on the positive-ion HRESIMS peak at *m*/*z* 343.1546 [M + H]^+^ (calcd for C_20_H_23_O_5_ 345.1540). The 1D NMR data of **5** showed similarities to those of **4** except for the chemical shifts of C-10′ (**5**: δ_H_ 1.37, δ_C_ 20.0; **4**: δ_H_ 1.60, δ_C_ 32.2) and C-2′ (**5**: δ_C_ 148.6; **4**: δ_C_ 142.3) (Tables 2 and 3). The stereochemical differences were studied by NOESY experiment. The key NOE interactions between H-3/H-2′, H-3/H-1′a, and H-3/H-4′α indicated H-3 and the vinyl group to be in an α orientation, and the NOESY cross-peaks between H-4′β/H_3_-10′ implied the β-orientation of the C-10′ methyl group (Figure 3). Therefore, compound **5** was assigned as a diastereomer of **4**, differing in position C-3′.

The isolated compounds **1**–**5** were investigated for their antiproliferative activity against a panel of human gynecological malignancies containing cells isolated from breast (MCF-7 and MDA-MB-231), cervical (HeLa and SiHa), and ovarian (A2780) cancers (Table S2, Supporting Information). Two concentrations (10 and 30 μM) were applied. The anticancer agent cisplatin was used as a reference agent. All compounds were deemed as inactive (IC_50_ < 10 μM) for the cancer cells used, and none were comparable to cisplatin. Only pauciflorins A (**1**), C (**3**), and D (**4**) elicited more than 30% cell growth inhibition against cervical cancer cells.

In conclusion, pauciflorins A–E (**1**–**5**) are hybrid molecules of chromone–monoterpene origin with unprecedented carbon skeletons. Pauciflorins A–C (**1**–**3**) can be derived by the connection of a 2-nor-chromone and a monoterpene unit, while pauciflorins D (**4**) and E (**5**) are dihydrochromone–monoterpene adducts with a rarely occurring orthoester functionality. Pauciflorin A (**1**) may be a biogenetic precursor of pauciflorin B (**2**). No experimental data were produced on the biosynthesis of 5-methylchromones or chromone-meroterpenoids. However, structurally related 5-methylcoumarins are biosynthesized via a polyketide intermediate from one acetate and four malonate units,^13^ with the same pathway having been found for both higher plants and fungi.^14^ A gerbera 2-pyrone synthase (G2PS)-type enzyme was found to be responsible for the formation of 4-hydroxy-5-methylcoumarin.^13^ A similar route could form the isomeric 2-hydroxy-5-methylchromone, which may be a critical biosynthetic intermediate for building chromone–monoterpene metabolites.

## Experimental Section

### General Experimental Procedures

The optical rotations were determined using a JASCO P-2000 polarimeter (JASCO International Co. Ltd., Hachioji, Tokyo, Japan). The NMR spectra were recorded in CDCl_3_ on a Bruker Avance DRX 500 spectrometer at 500 MHz (^1^H) and 125 MHz (^13^C). The signals of the deuterated solvents were taken as references. Two-dimensional (2D) NMR experiments were performed using standard Bruker software. Gradient-enhanced versions were applied in the COSY, HSQC, and HMBC experiments. The HRESIMS spectra were acquired using a Thermo Scientific Q-Exactive Plus Orbitrap mass spectrometer equipped with an ESI ion source using the positive-ionization mode. The data were acquired and processed using MassLynx software. Vacuum-liquid chromatography (VLC) was performed on silica gel (15 μm, Merck), and LiChroprep RP-18 (40–63 μm, Merck) stationary phase was used for reversed-phase VLC, while open column chromatography (CC) was conducted on polyamide (MP Biomedicals). Preparative thin-layer chromatography (TLC) was carried out on silica gel 60 F_254_ plates (Merck). High-performance liquid chromatography (HPLC) was carried out on a Wufeng HPLC, Waters HPLC, and an Agilent HPLC, using normal [LiChrospher Si 60 (4 × 250 mm, 5 μm) and Luna (R) Silica (2) 100 l (250 × 21.2 mm, 5 μm)] and reversed-phase [Kinetex C_18_ 100A (4.6 × 150 mm, 5 μm) and Agilent Zorbax ODS C_18_ 100A (9.4 × 250 mm, 5 μm)] columns. The TLC plates were visualized under a UV lamp at 254 nm and detected by spraying with concentrated sulfuric acid, followed by heating. All solvents used for CC and TLC were at least of analytical grade (VWR Ltd., Hungary).

### Plant Material

The aerial parts of *Centrapalus pauciflorus* were collected in August 2018 in Zaria, Nigeria (11°7′19.758″ N 7°43′23.1672″ E) and were identified by Umar Shehu Gallah (National Research Institute for Chemical Technology, NARICT), Zaria, Nigeria. Voucher specimens were deposited at NARICT under the number Narict/Biores/321, and in the herbarium, Department of Pharmacognosy, University of Szeged, Szeged, Hungary, number 897.

### Extraction and Isolation

The air-dried and powdered plant material (548 g) was extracted by percolation with methanol (45 L) at room temperature. The MeOH extract was concentrated (133 g), dissolved in 1000 mL of MeOH–H_2_O (1:1), and subjected to solvent–solvent partitioning with CHCl_3_ (3 × 1000 mL) to give the organic phase. This chloroform phase (65.81 g) was subjected to polyamide open CC (250 g) with MeOH–H_2_O (1:4, 2:3, 3:2, 4:1, and 5:0) mixtures as eluents. Five major fractions were collected according to the eluents. The fraction obtained using MeOH–H_2_O (3:2) (14 g) was separated further by VLC on silica gel using gradient elution with cyclohexane–EtOAc–EtOH (9:1:0, 8:2:0, 7:3:0, 50:20:1.5, 50:20:3, 50:20:6, 50:20:9, 50:20:12, 50:20:15, 5:2:2, 5:2:4, 5:2:6, and 5:2:8). The fractions collected were monitored by TLC. Those with similar profiles were combined, giving nine fractions, A–I. Fractions A obtained from cyclohexane–EtOAc–EtOH (9:1 and 8:2) was rechromatographed by normal-phase VLC (NP-VLC) on silica gel with cyclohexane–EtOAc gradient mixtures, yielding two subfractions, A/I and A/II. Subfraction A/I was purified further by reversed-phase HPLC (RP-HPLC), affording nine fractions, A/I/1–9. Further purification of fraction A/I/3 on NP-HPLC with *n*-hexane–EtOAc (8:2) as the mobile phase furnished compounds **2** (1.5 mg) and **3** (1.1 mg). RP-HPLC of fraction A/II gave five subfractions (A/II/1–5), of which one, A/II/4, contained the pure compound **5** (6.9 mg). NP-HPLC purification of subfraction A/II/2 with *n*-hexane–EtOAc as the mobile phase resulted in the isolation of compound **4** (1.1 mg). Fraction C was subjected to reversed-phase flash column chromatography (RP-FCC) on silica gel with MeOH–H_2_O mixtures (70:30 to 100:0 gradient slope for 2 h) as eluents to obtain seven subfractions, C/I–VII. Fraction C/III was chromatographed by NP-VLC on silica gel with *n*-hexane–CHCl_3_ mixtures (10:0, 9:1, 8:2, 7:3, 6:4, 5:5, 4:6, 3:7, 2:8, and 0:10), which gave five subfractions, C/III/1–5. White crystals were observed in fractions C/III/2–4 after storage in the refrigerator. The crystals were removed by filtration and purified further by recrystallization, affording compound **1**. The mother liquors of the crystals were subjected to NP-HPLC using *n*-hexane–EtOAc–MeOH (95:4:1) as the mobile phase and then to RP-HPLC with MeOH–H_2_O (70:30) as the eluent, furnishing more compound **1** (5.0 mg).

#### Pauciflorin A (**1**)

Colorless oil; [α]^25^_D_ −157.9 (*c* 0.1, CHCl_3_); ^1^H and ^13^C NMR data, see Tables 1 and 2; HRESIMS *m*/*z* 339.1567 [M + Na]^+^ (calcd for C_19_H_24_O_4_Na^+^, 339.1567), 299.1640 [M – H_2_O + H]^+^ (calcd for C_19_H_23_O_3_, 299.1642).

#### Pauciflorin B (**2**)

White amorphous powder; [α]^27^_D_ −270.8 (*c* 0.1, CHCl_3_); ^1^H and ^13^C NMR data, see Tables 1 and 2; positive-ion HRESIMS *m*/*z* 299.1641 [M + H]^+^ (calcd for C_19_H_23_O_3_^+^, 299.1642), 321.1461 [M + Na]^+^ (calcd for C_19_H_22_O_3_Na, 321.1642).

#### Pauciflorin C (**3**)

White amorphous powder; [α]^27^_D_ +67.6 (*c* 0.05, CHCl_3_); ^1^H and ^13^C NMR data, see Tables 1 and 2; HRESIMS *m*/*z* 299.1646 [M + H]^+^ (calcd for C_19_H_23_O_3_^+^, 299.1642).

#### Pauciflorin D (**4**)

Colorless oily material; [α]^26^_D_ +6.9 (*c* 0.05, CHCl_3_); ^1^H and ^13^C NMR data, see Tables 1 and 2; HRESIMS *m*/*z* 343.1539 [M + H]^+^ (calcd for C_20_H_23_O_5_^+^, 343.1540), 365.1358 [M + Na]^+^ (calcd for C_20_H_22_O_5_Na^+^, 365.1359).

#### Pauciflorin E (**5**)

Colorless oily material; [α]^27^_D_ +21.9 (*c* 0.1, CHCl_3_); ^1^H and ^13^C NMR data, see Tables 1 and 2; HRESIMS *m*/*z* 343.1546 [M + H]^+^ (calcd for C_20_H_23_O_5_^+^, 345.1540).

### X-ray Crystallography of Compound **1**

The absolute configuration of **1** was determined by X-ray crystallography (Figure 4). The colorless single crystals grown from a mixture of methanol and ethyl acetate at −5 °C were light and thermally sensitive. They crystallized in the trigonal system, space group *P*3_1_2_1_. The absolute configuration of the chiral atoms was C-3 (*R*), C-3′ (*S*), and C-6′ (*S*), Flack *x* = 0.11(5), Parsons *z* = 0.09(5). Details of molecular conformation, intra-, and intermolecular interactions, and packing arrangement can be found in the Supporting Information.

Crystallographic data of compound **1** have been deposited in the Cambridge Crystallographic Data Centre with the deposition number CCDC 2224099. Copies of the data can be obtained free of charge via www.ccdc.cam.ac.uk/data_request/cif, or by emailing data_request@ccdc.cam.ac.uk, or by contacting the Cambridge Crystallographic Data Centre, 12 Union Road, Cambridge CB2 1EZ, UK [fax: + 44.(0)1223 336033].

#### Crystallographic data of (2*S*,3*R*,4*S*)-3-(2-hydroxy-6-methylbenzoyl)-2-(2-hydroxypropan-2-yl)-4-methyl-4-vinylcyclopentanone (**1**)

Intensity data were collected on an RAXIS-RAPID II diffractometer (graphite monochromator; Cu Kα radiation, λ = 1.54178 Å). C_19_H_24_O_4_, *M*_r_ = 316.38, size 0.5 × 0.4 × 0.4 mm, *a* = 10.0520(3)Å, *b* = 10.0520(3)Å, *c* = 29.6147(9) Å, α = 90°, β = 90°, γ = 120°, *V* = 2591.45(17) Å^3^, trigonal, space group *P*3_1_21, *Z* = 6, *T* = 103(2) K, absorption coefficient 0.681 mm^–1^, numerical absorption correction (*T*_min_ = 0.94800, *T*_max_ = 0.95530), *F*(000) = 1020, θ for data collection 4.479–68.251°, index ranges −12 ≤ *h* ≤ 11, −12 ≤ *k* ≤ 12, −35 ≤ *l* ≤ 35, 62 994 reflections collected, 3155 independent reflections [*R*_(int)_ = 0.0773] and [*R*_(sigma)_ = 0.0238], completeness to θ = 68.251° (99.9%), data/restraints/parameters 3155/0/222, largest diff peak and hole 0.17 and −0.13 e A^–3^. The final *R*_1_ = 0.0344 [*I* > 2σ(*I*)], final *wR*_2_ = 0.0865. The final *R*_1_ (all data) = 0.0337, *wR*_2_ (all data) = 0.0871. The goodness of fit on *F*^2^ = 1.069. The absolute structure parameter is 0.11(5), Friedel coverage: 1.000.

### Determination of Antiproliferative Properties

The effects of compounds **1**–**5** on the growth of a panel of human adherent tumor cell lines were determined using an MTT (3-(4,5-dimethylthiazol-2-yl)-2,5-diphenyltetrazolium bromide) assay.^15^ The cell lines isolated from cervical (HeLa), breast (MCF-7 and MDA-MB-231), and ovarian cancers (A2780) were obtained from the European Collection of Cell Cultures (Salisbury, UK), while the SiHa cervical tumor cell line was purchased from the American Tissue Culture Collection (Manassas, VA, USA). All the cells were cultivated in minimal essential medium supplemented with fetal bovine serum (10%), nonessential amino acids (1%), and penicillin–streptomycin (1%) at 37 °C in a humidified atmosphere containing 5% CO_2_. All media and supplements were purchased from Lonza Group Ltd. (Basel, Switzerland). The cells were plated into 96-well plates at a density of 5000 cells/well. After overnight incubation, the tested molecules were added at two final concentrations (10 and 30 μM). After incubation for 72 h, an MTT solution (20 μL, 5 mg/mL) was added to each well and incubated further for four h. Finally, the medium was removed, and the formazan produced was dissolved in DMSO for 60 min of shaking at 37 °C. The absorbance was determined at 545 nm using a microplate reader (SpectoStarNano, BMG Labtech, Ortenberg, Germany).

## Supporting Information Available

HRESIMS, NMR spectra, and single-crystal X-ray diffraction data for the isolated compounds; data of antiproliferative assay (PDF)
X-ray crystallographic data for **1** (CIF)

## Supplementary Material

np2c01132_si_001.pdf

np2c01132_si_002.cif
