# Supplementary material for: Pauciflorins A–E, Unexpected Chromone–Monoterpene-Derived Meroterpenoids from Centrapalus pauciflorus
Source: J Nat Prod. 2023 Mar 18;86(4):891–6. doi: 10.1021/acs.jnatprod.2c01132 (PMC10152445; doi:10.1021/acs.jnatprod.2c01132)
Supplement: Supplementary file 1 — np2c01132_si_001.pdf [file np2c01132_si_001.pdf]

SUPPORTING INFORMATION

Pauciflorins A–E, Unexpected Chromone-Monoterpene Derived Meroterpenoids from *Centrapalus pauciflorus*

Gordana Krstić,[†,‡,#] Muhammad Bello Saidu,[†,#] Petra Bombicz,[§] Sourav De,[§] Hazhmat Ali,[⊥] István Zupkó,[⊥] Róbert Berkecz,[∥] Umar Shehu Gallah,[▽] Dóra Rédei,[†] Judit Hohmann[*,†,○]

[†]Department of Pharmacognosy, University of Szeged, Eötvös u. 6, 6720 Szeged, Hungary

[‡]University of Belgrade - Faculty of Chemistry, Studentski trg 12-16, 11158 Belgrade, Serbia

[§]Centre for Structural Science, Research Centre for Natural Sciences, Magyar Tudósok körútja 2, 1117 Budapest, Hungary

[⊥]Institute of Pharmacodynamics and Biopharmacy, University of Szeged, Eötvös u. 6, 6720 Szeged, Hungary

[∥]Institute of Pharmaceutical Analysis, University of Szeged, Somogyi u. 4, 6720 Szeged, Hungary

[▽]Bioresource Department, National Research Institute for Chemical Technology (NARICT), Zaria, Nigeria

[○]ELKH-USZ Biologically Active Natural Products Research Group, University of Szeged, Eötvös u. 6, H-6720 Szeged, Hungary

Content

1. Antiproliferative properties of the fractions and compounds (**1**–**5**) (Tables S1, S2) 2

2. NMR and mass spectra of compounds **1**–**5** 3

3. Structure determination of compound **1** by single crystal X-ray diffraction 22

9. References 33

1. Antiproliferative properties of the fractions and compounds **1**–**5** by MTT assay [1]

Table S1. Antiproliferative activity of the fractions obtained from chloroform extract of *Centrapalus pauciflorus*, measured by MTT assay on A2780, MDA-MB-231, HeLa, and MCF-7 tumor cell lines in 10 and 30 μg/mL.

Fraction	Conc. (μg/mL)	Cell growth inhibition (%) ± SEM MCF-7	MDA-MB-231	HeLa	A2780
**1**	10	**57.2** ± 0.6	29.4 ± 1.2	**48.2** ± 0.8	5.0 ± 0.7
	30	**68.1** ± 0.8	**50.1** ± 0.8	**61.5** ± 0.9	24.1 ± 1.0
**2**	10	30.0 ± 1.7	31.1 ± 3.0	27.8 ± 1.9	–
	30	33.2 ± 1.6	40.8 ± 2.1	7.8 ± 1.1	30.0 ± 1.2
**3**	10	**44.2** ± 0.6	**49.3** ± 0.9	**54.6** ± 0.6	5.2 ± 0.5
	30	**70.7** ± 0.4	**85.3** ± 1.0	**63.7** ± 1.3	**68.2** ± 0.8
**4**	10	12.6 ± 0.8	29.0 ± 1.2	13.5 ± 0.6	–
	30	17.3 ± 0.6	42.1 ± 0.8	13.6 ± 2.0	–
**5**	10	7.9 ± 1.1	18.2 ± 1.3	16.9 ± 0.7	–
	30	9.3 ± 1.0	28.4 ± 1.8	17.1 ± 1.2	–

Table S2. Antiproliferative properties of compounds **1**–**5**

Compound	Conc. (μM)	Cell growth inhibition (%) ± SEM MCF-7	MDA-MB-231	HeLa	SiHa	A2780
Pauciflorin A (**1**)	10	–*	–	–	–	–
	30	–	–	40.76 ± 3.71	–	–
Pauciflorin B (**2**)	10	12.32 ± 1.93	–	–	10.36 ± 2.36	–
	30	29.64 ± 2.09	–	13.67 ± 1.52	28.70 ± 1.13	–
Pauciflorin C (**3**)	10	13.37 ± 2.22	–	–	20.81 ± 1.53	–
	30	29.56 ± 2.36	–	15.71 ± 2.22	39.27 ± 1.32	–
Pauciflorin D (**4**)	10	–	–	–	–	–
	30	–	–	33.42 ± 1.80	12.06 ± 1.28	23.32 ± 1.68
Pauciflorin E (**5**)	10	–	11.97 ± 2.59	23.51 ± 1.86	17.18 ± 3.07	–
	30	15.28 ± 3.14	16.19 ± 2.42	25.40 ± 2.16	21.51 ± 1.58	–
cisplatin	10	66.91 ± 1.81	42.72 ± 2.68	42.61 ± 2.33	60.98 ± 0.92	83.57 ± 2.21
	30	96.80 ± 0.35	86.44 ± 0.42	99.93 ± 0.26	88.95 ± 0.53	95.02 ± 0.28

* Cell growth inhibition values less than 10% were considered negligible and are not given numerically

2. NMR and mass spectra of compounds **1**–**5**

S1. ^{1}H NMR spectrum of **1**
S2. ^{13}C NMR spectrum of **1**
S3. HSQC spectrum of **1**
S4. HMBC spectrum of **1**
S5. COSY spectrum of **1**
S6. NOESY spectrum of **1**
S7. Mass spectrum of **1**
S8. ^{1}H NMR spectrum of **2**
S9. ^{13}C NMR spectrum of **2**
S10. HSQC spectrum of **2**
S11. HMBC spectrum of **2**
S12. COSY spectrum of **2**
S13. NOESY spectrum of **2**
S14. Mass spectrum of **2**
S15. ^{1}H NMR spectrum of **3**
S16. ^{13}C NMR spectrum of **3**
S17. HSQC spectrum of **3**
S18. HMBC spectrum of **3**
S19. COSY spectrum of **3**
S20. NOESY spectrum of **3**
S21. Mass spectrum of **3**
S21. ^{1}H NMR spectrum of **4**
S22. ^{13}C NMR spectrum of **4**
S23. HSQC spectrum of **4**
S24. HMBC spectrum of **4**
S25. COSY spectrum of **4**
S26. NOESY spectrum of **4**
S27. Mass spectrum of **4**
S28. ^{1}H NMR spectrum of **5**
S29. ^{13}C NMR spectrum of **5**
S30. HSQC spectrum of **5**
S31. HMBC spectrum of **5**
S32. COSY spectrum of **5**
S34. NOESY spectrum of **5**
S35. Mass spectrum of **5**

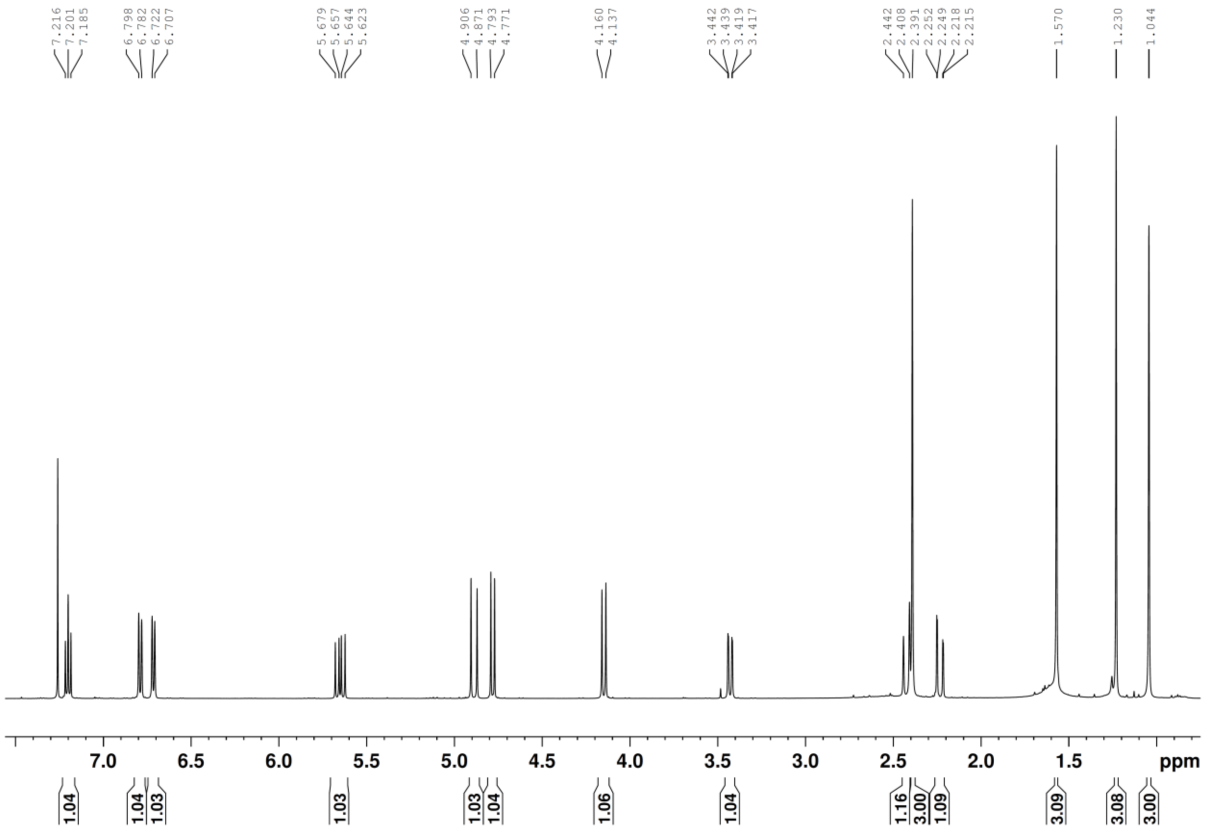

S1. ^{1}H NMR spectrum of **1**, $CDCl_3$, 298 K

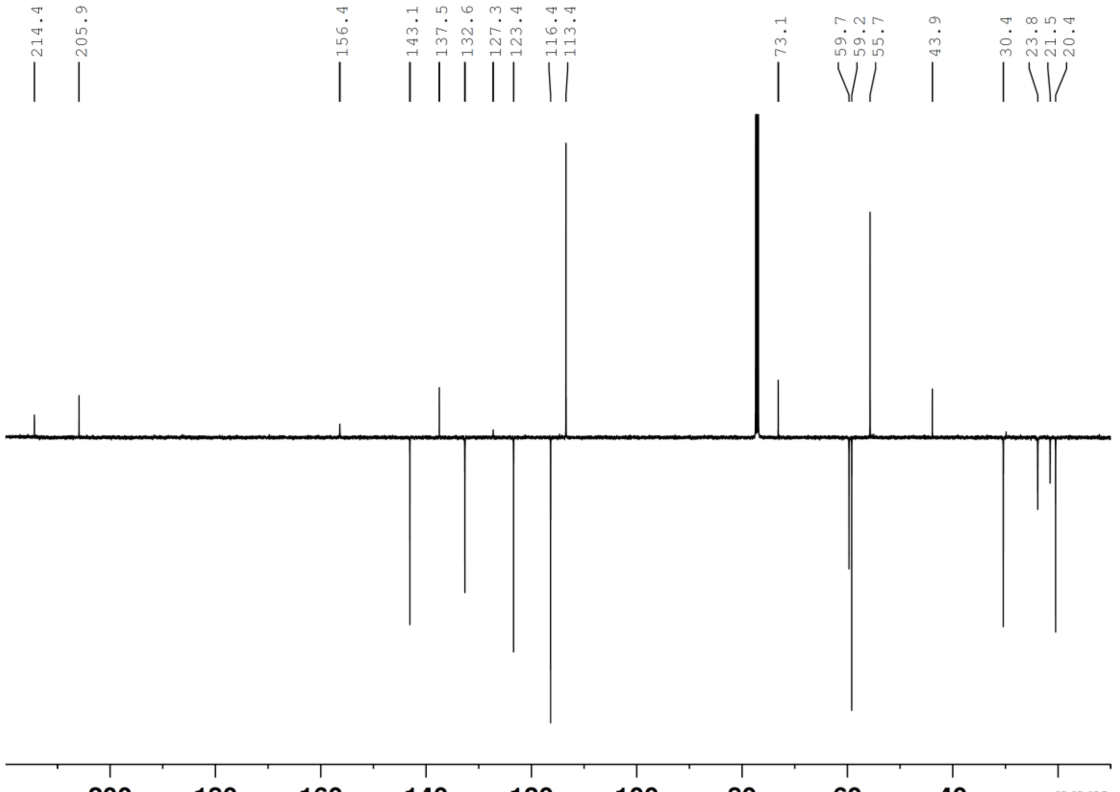

S2. ^{13}C NMR spectrum of **2**, $CDCl_3$, 298 K

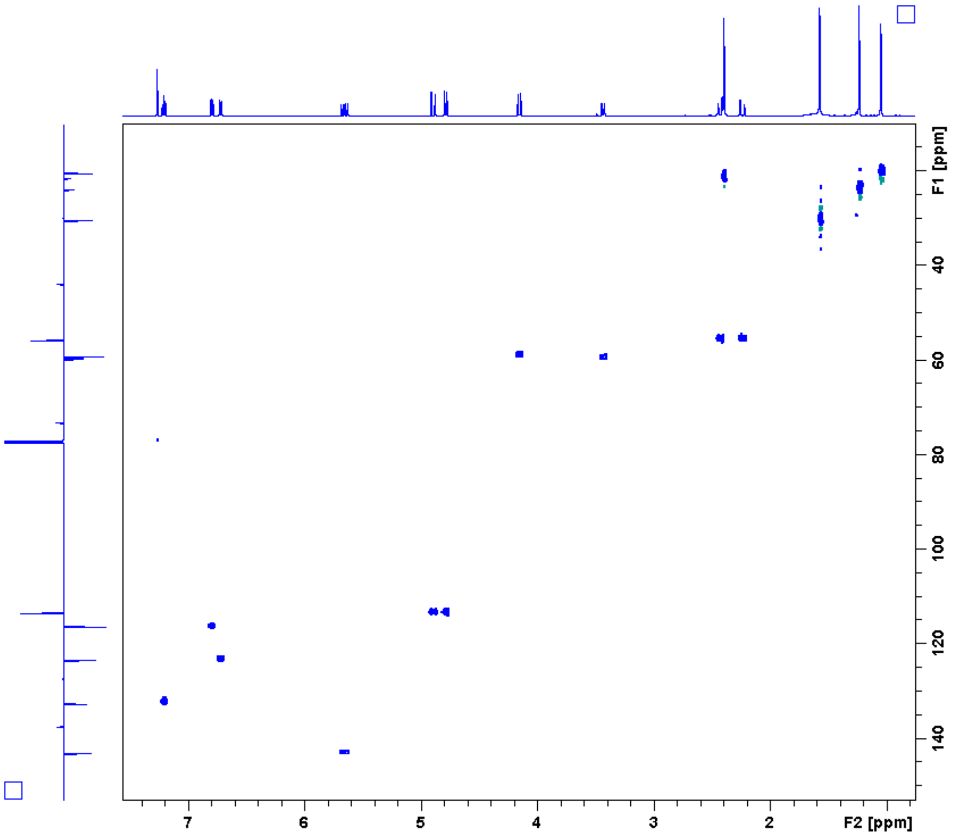

S3. HSQC spectrum of **1**, $CDCl_3$, 298 K

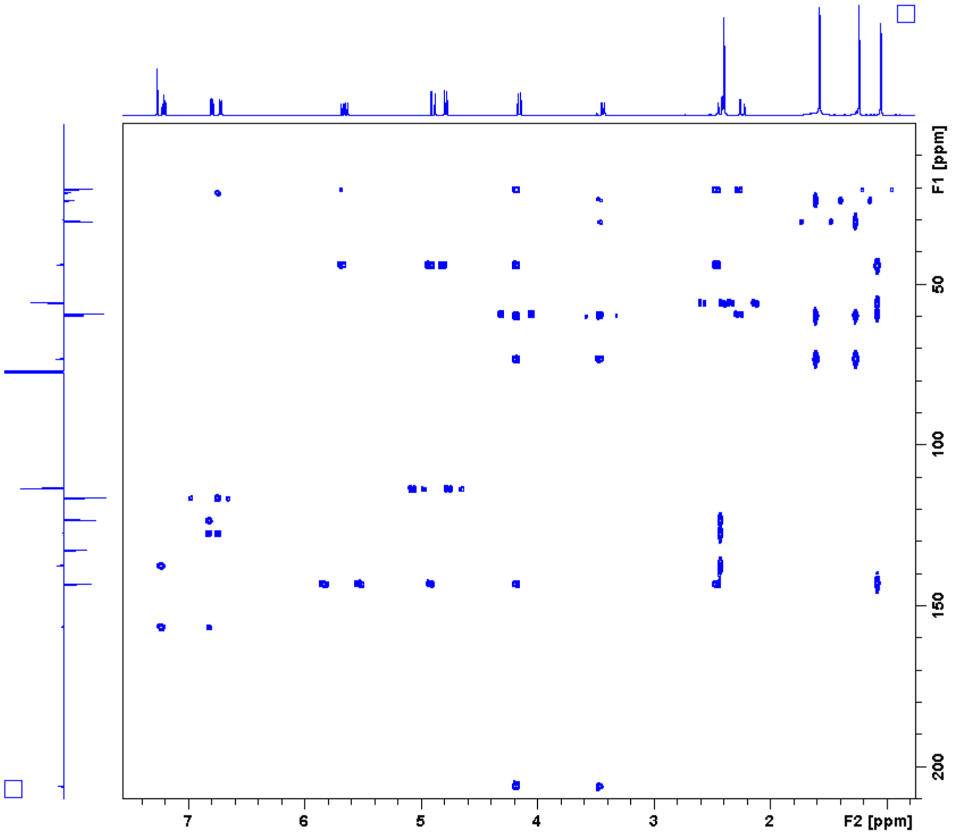

S4. HMBC spectrum of **1**, $CDCl_3$, 298 K

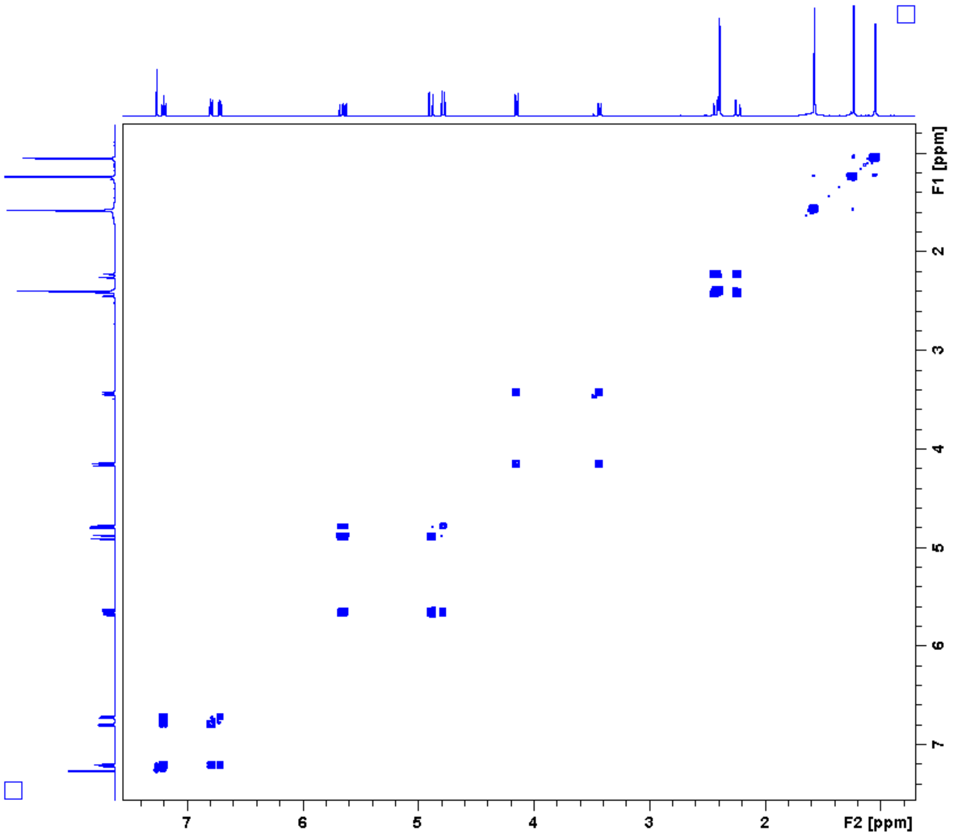

S5. COSY spectrum of **1**, $CDCl_3$, 298 K

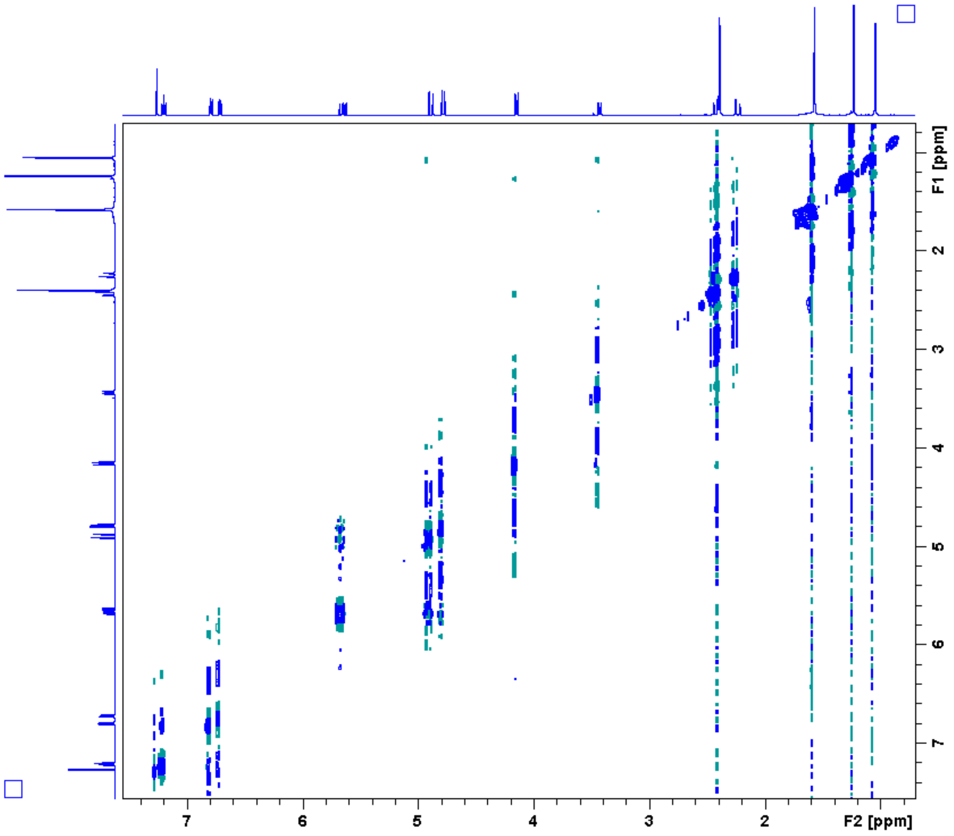

S6. NOESY spectrum of **1**, $CDCl_3$, 298 K

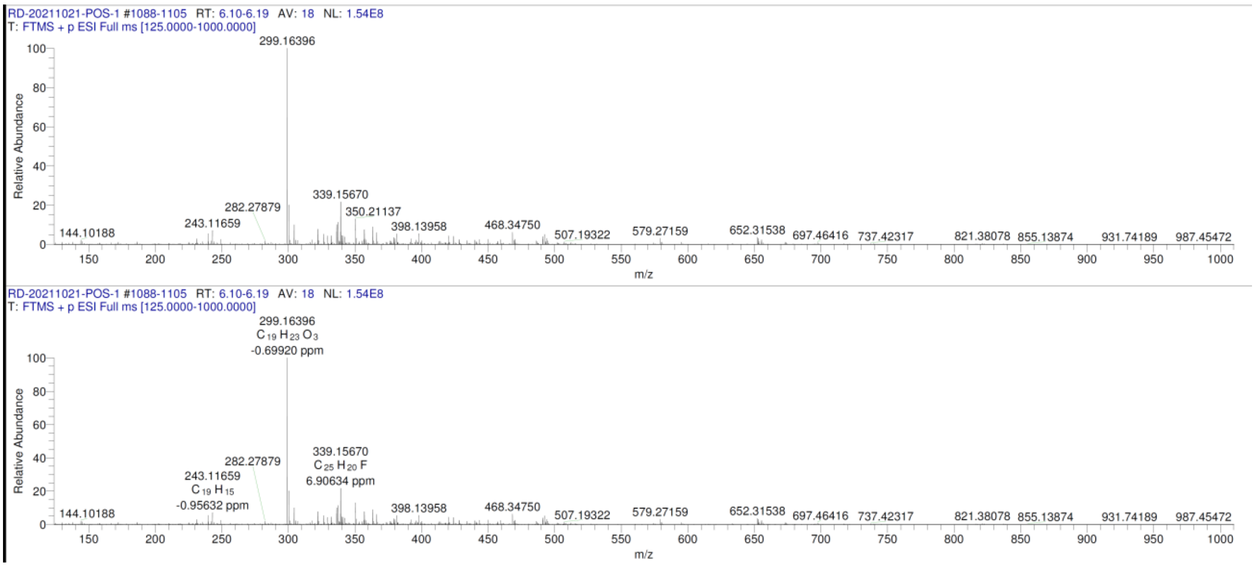

S7. Mass spectrum of **1**, $CDCl_3$, 298 K

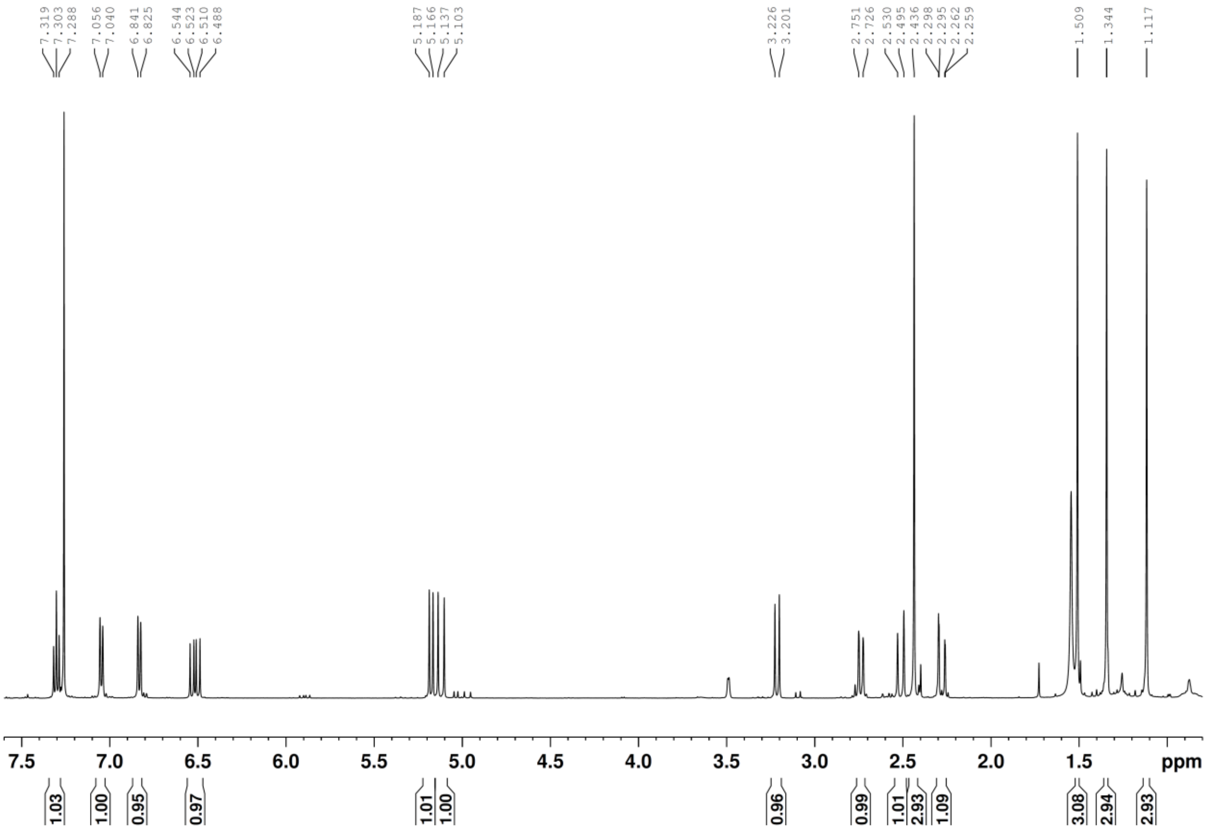

S8. ^{1}H NMR spectrum of **2**, $CDCl_3$, 298 K

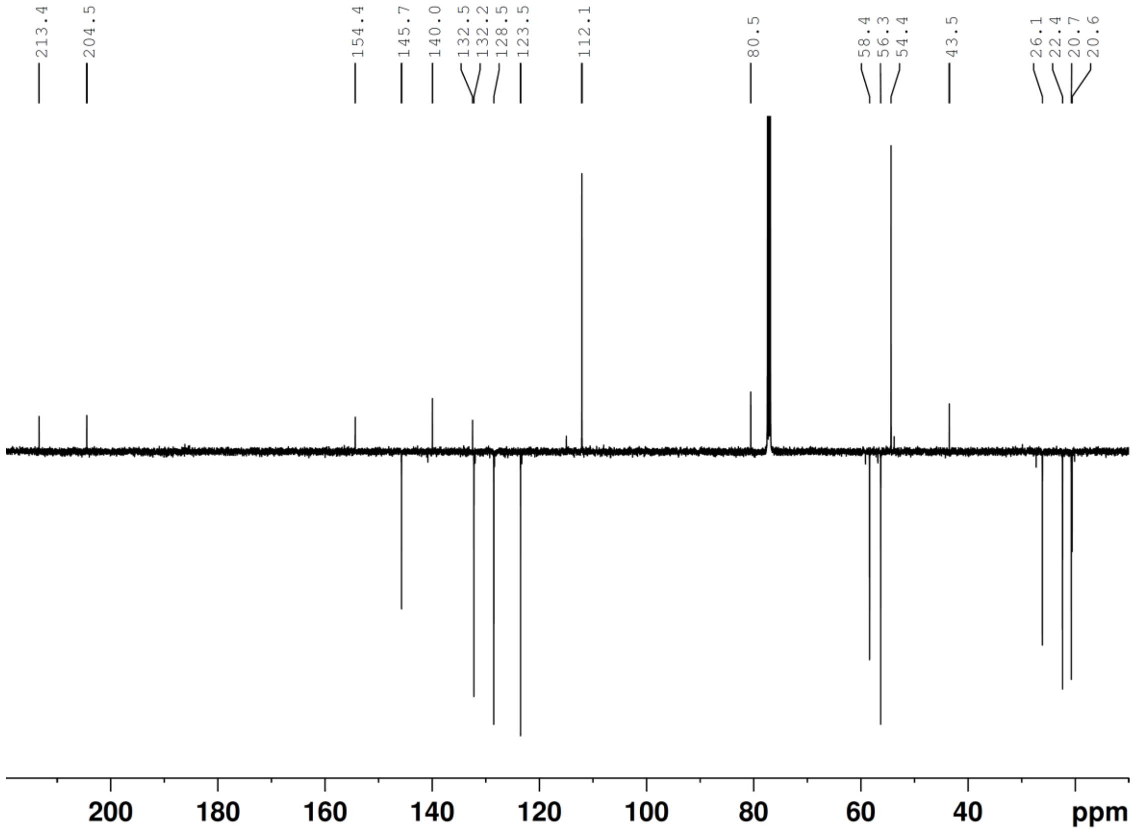

S9. ^{13}C NMR spectrum of **2**, $CDCl_3$, 298 K

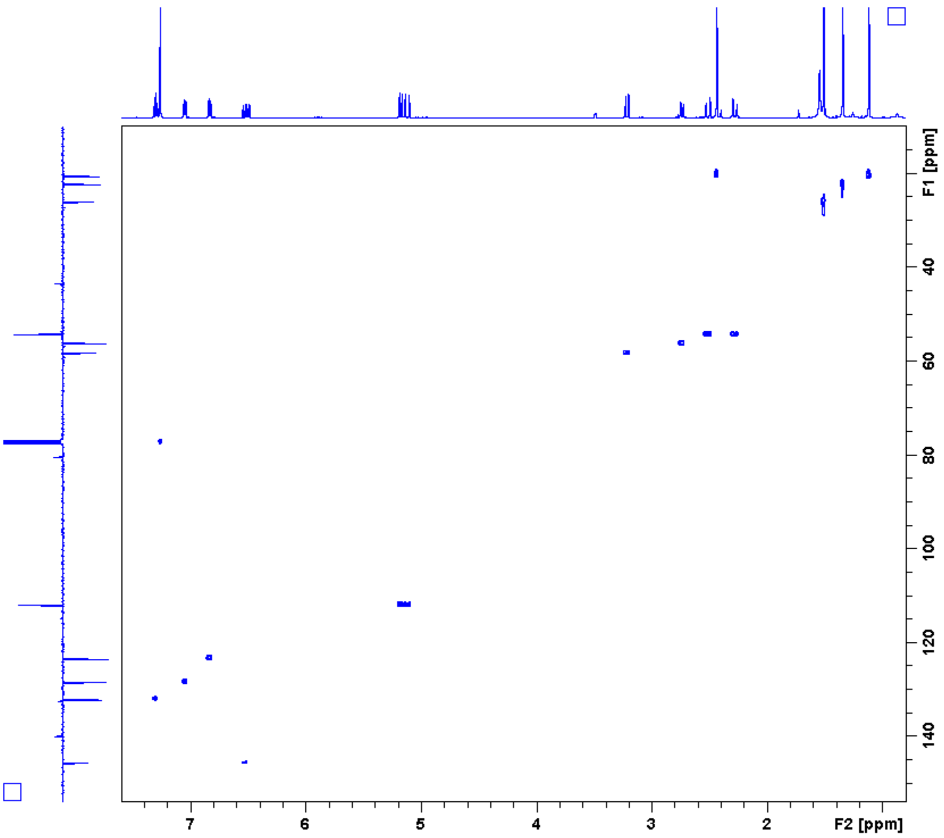

S10. HSQC spectrum of **2**, $CDCl_3$, 298 K

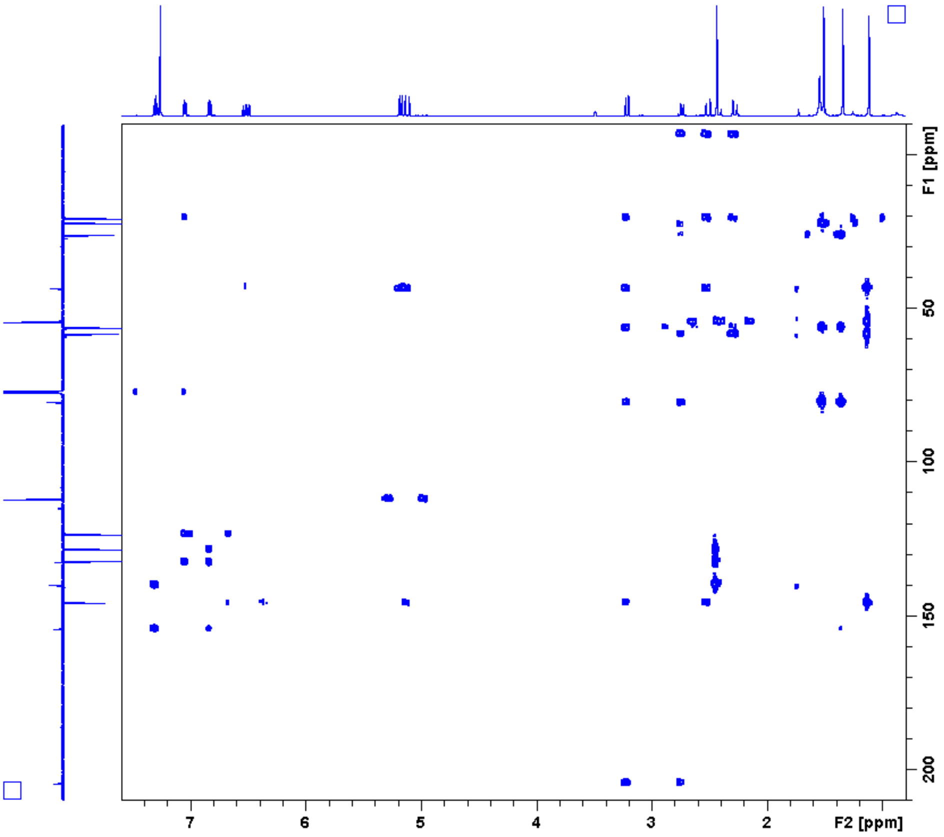

S11. HMBC spectrum of **2**, $CDCl_3$, 298 K

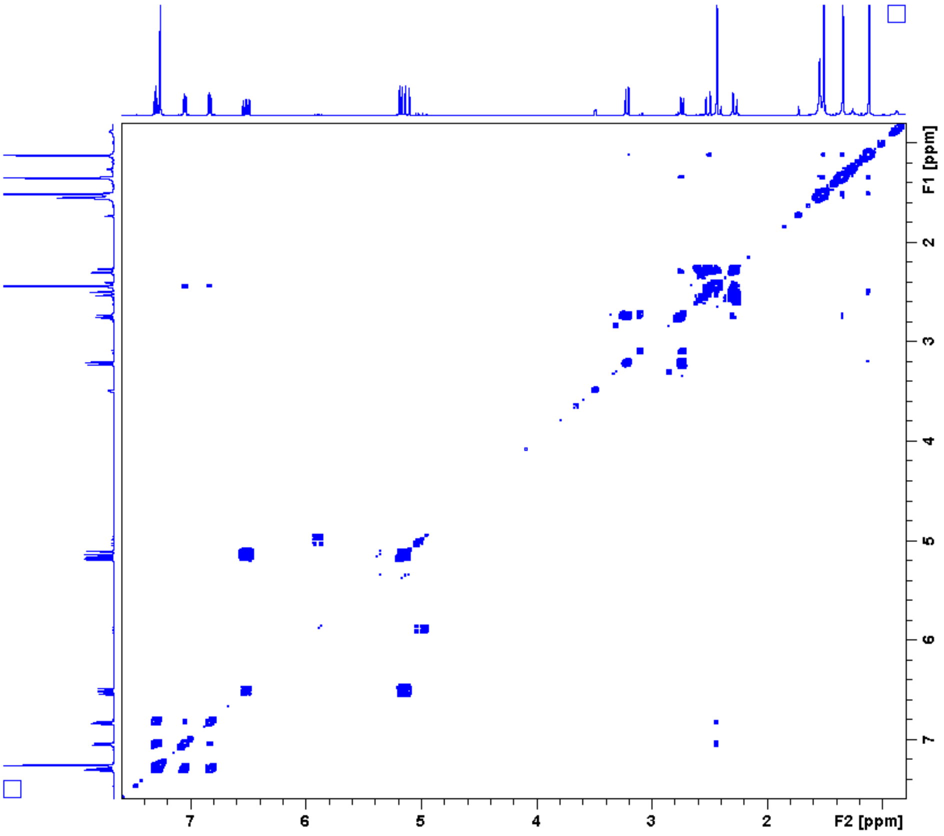

S12. COSY spectrum of **2**, $CDCl_3$, 298 K

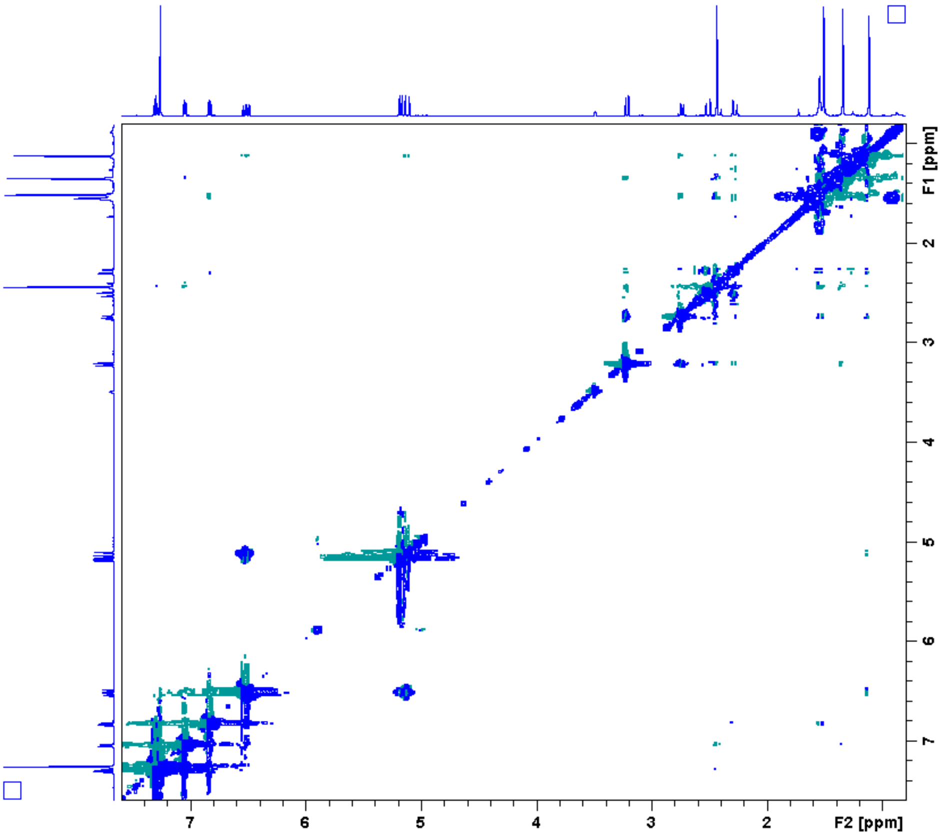

S13. NOESY spectrum of **2**, $CDCl_3$, 298 K

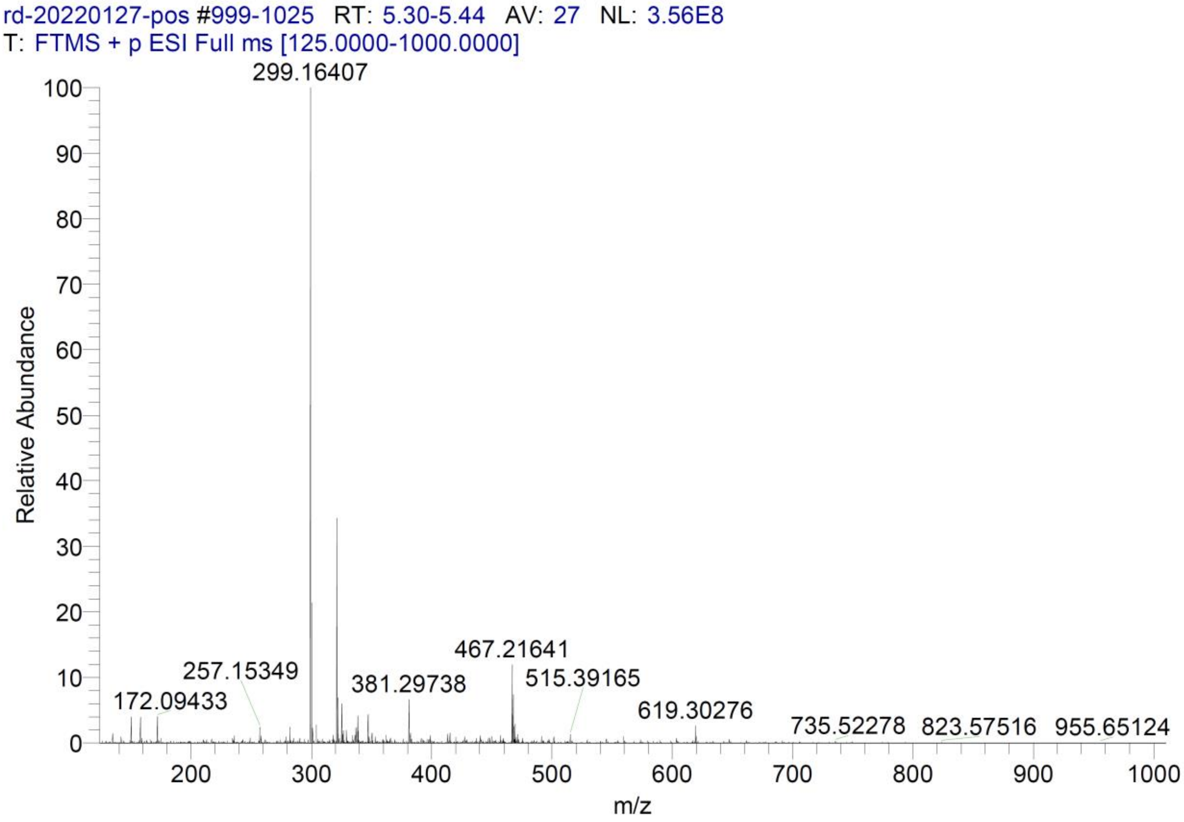

S14. Mass spectrum of **2**, positive mode

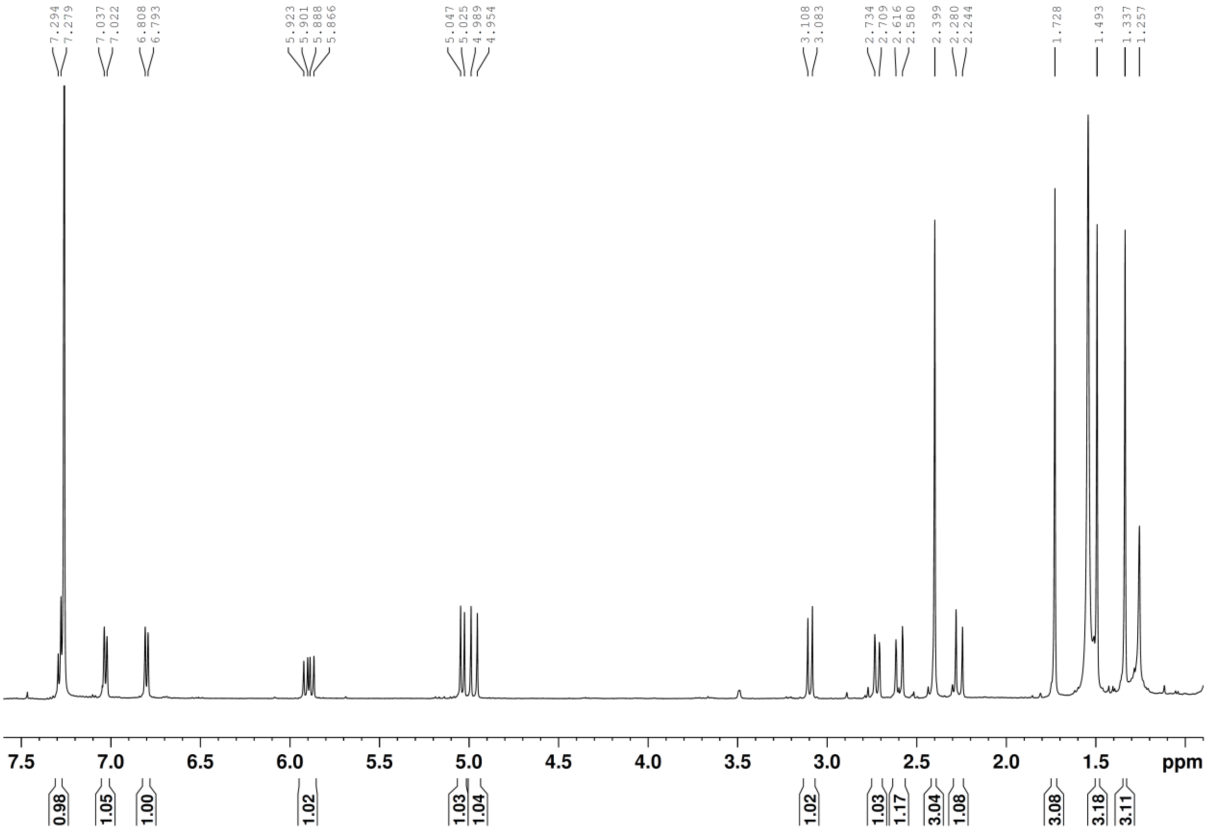

S15. ^{1}H NMR spectrum of **3**, $CDCl_3$, 298 K

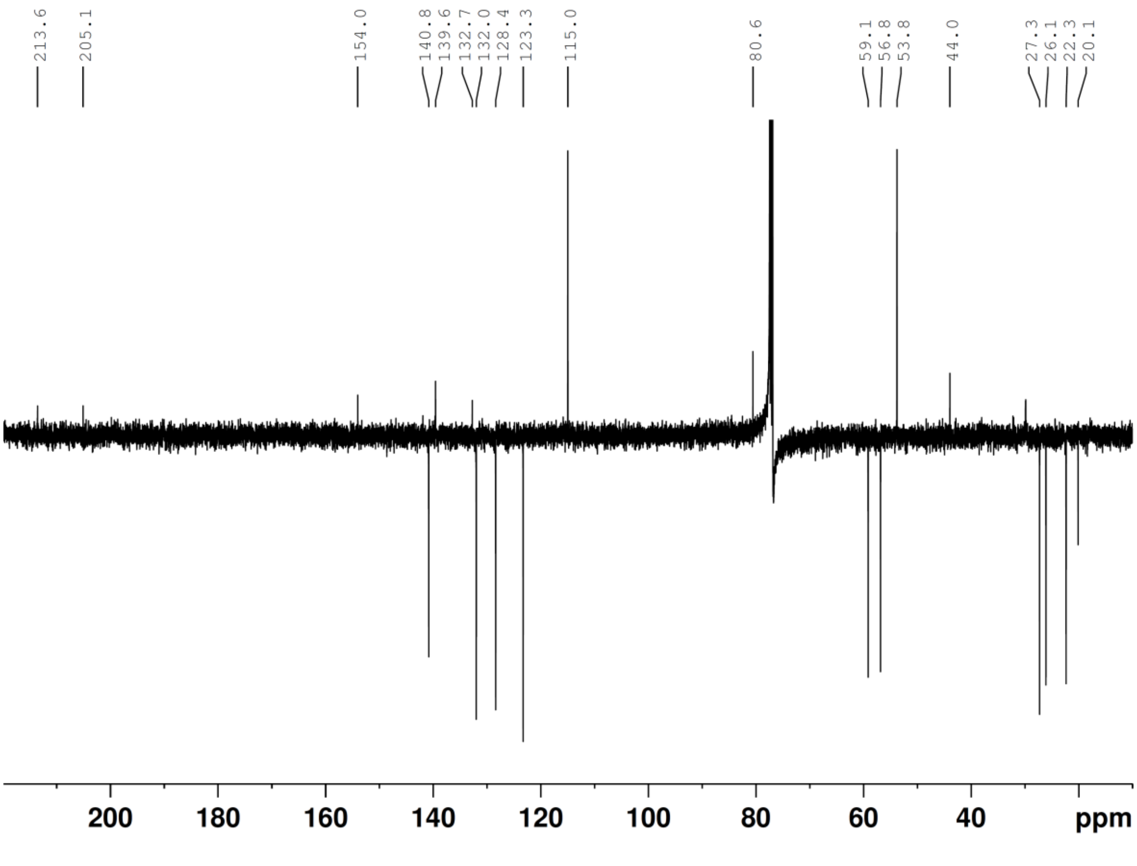

S16. ^{13}C NMR spectrum of **3**, $CDCl_3$, 298 K

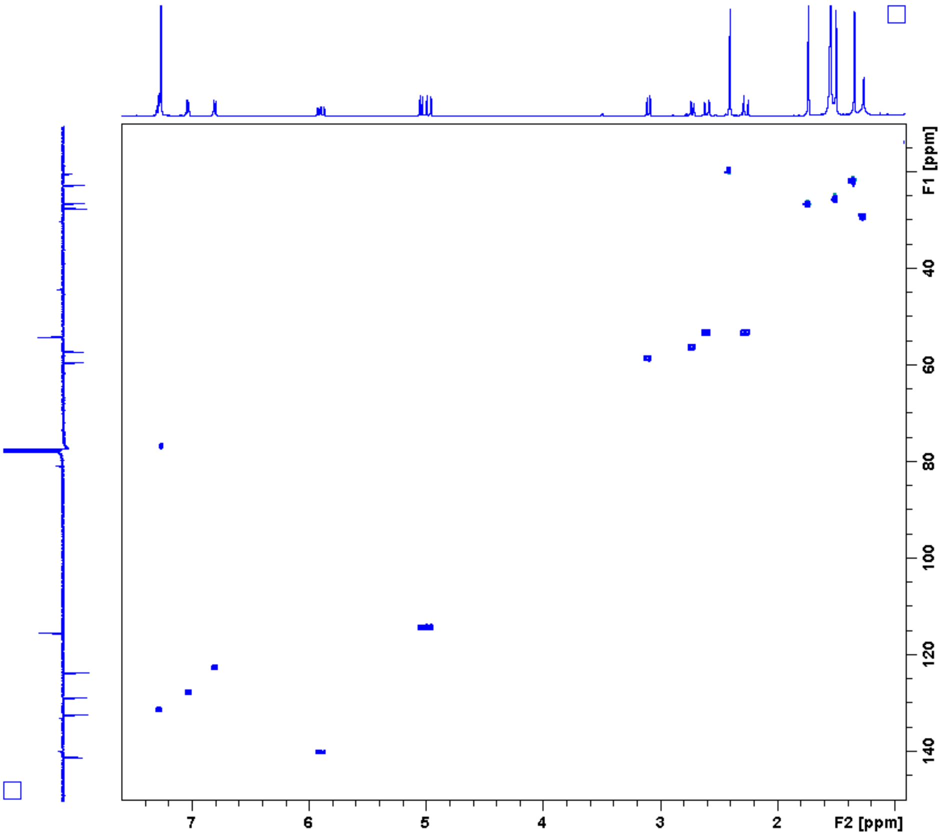

S17. HSQC spectrum of **3**, $CDCl_3$, 298 K

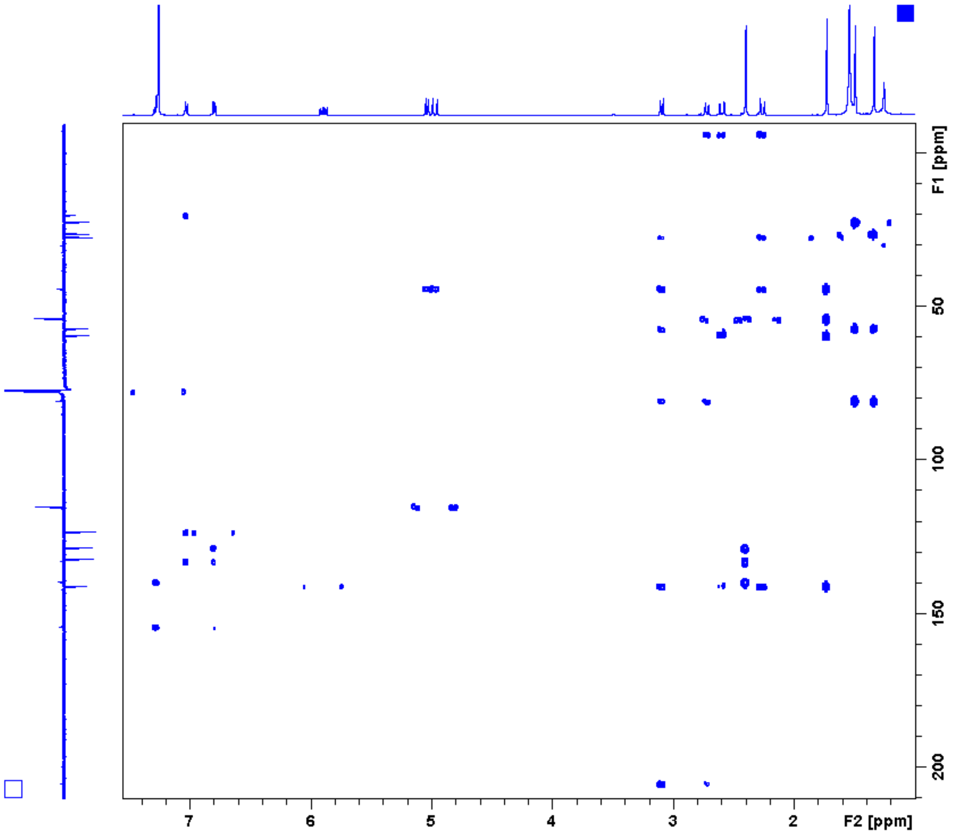

S18. HMBC spectrum of **3**, $CDCl_3$, 298 K

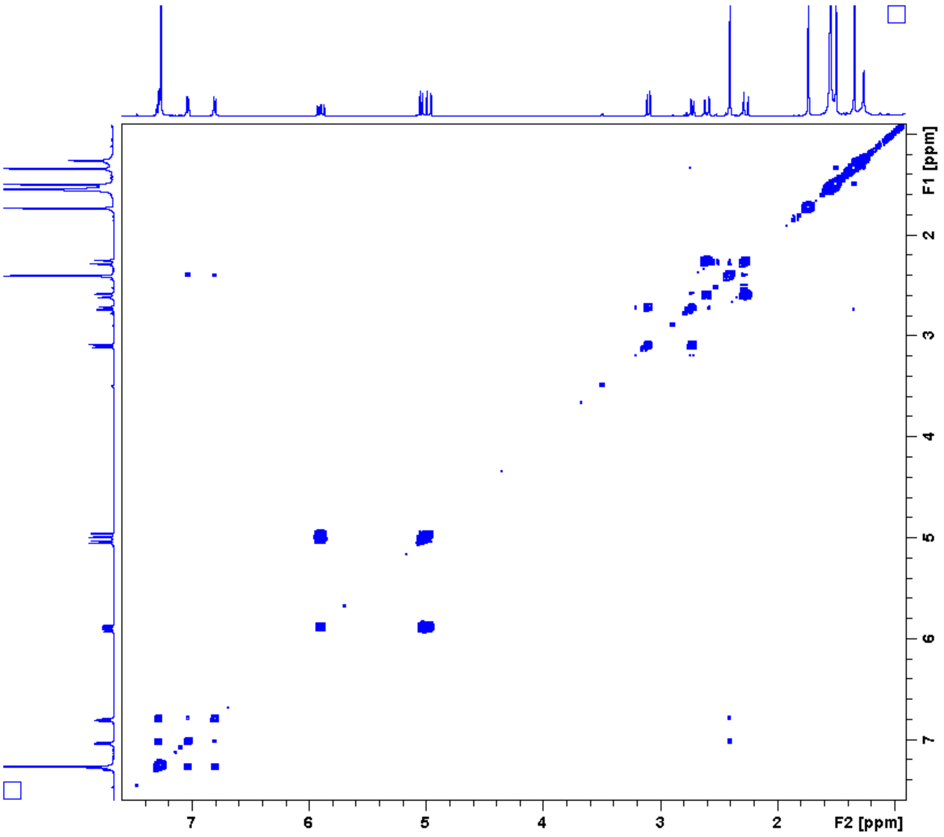

S19. COSY spectrum of **3**, $CDCl_3$, 298 K

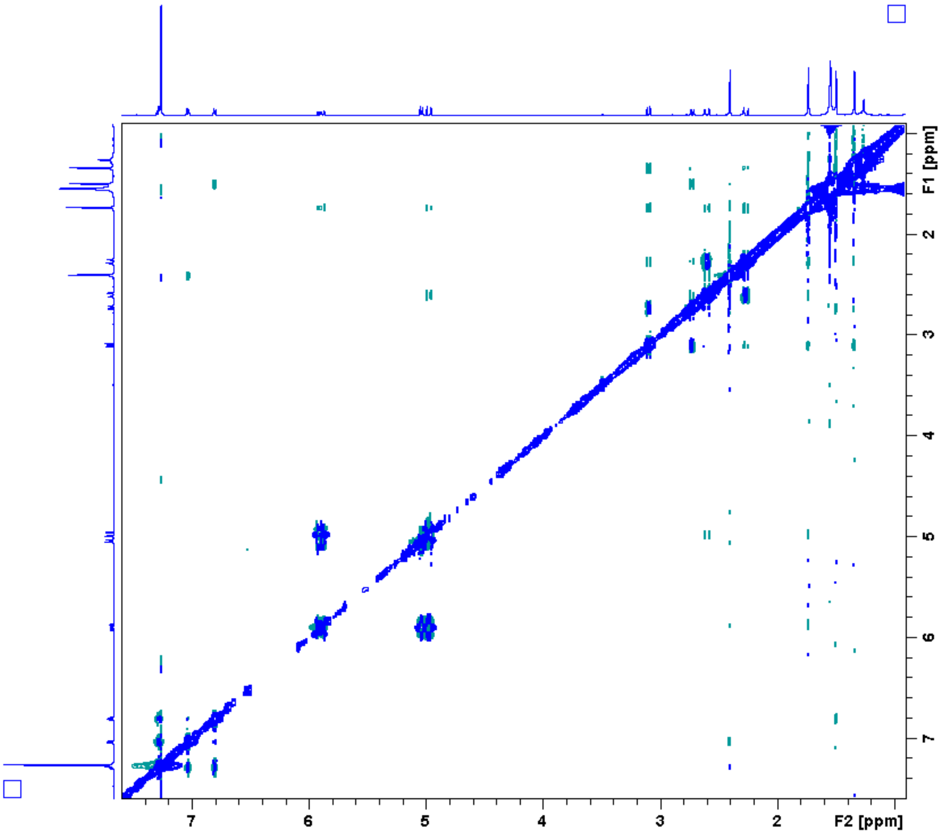

S20. NOESY spectrum of **3**, $CDCl_3$, 298 K

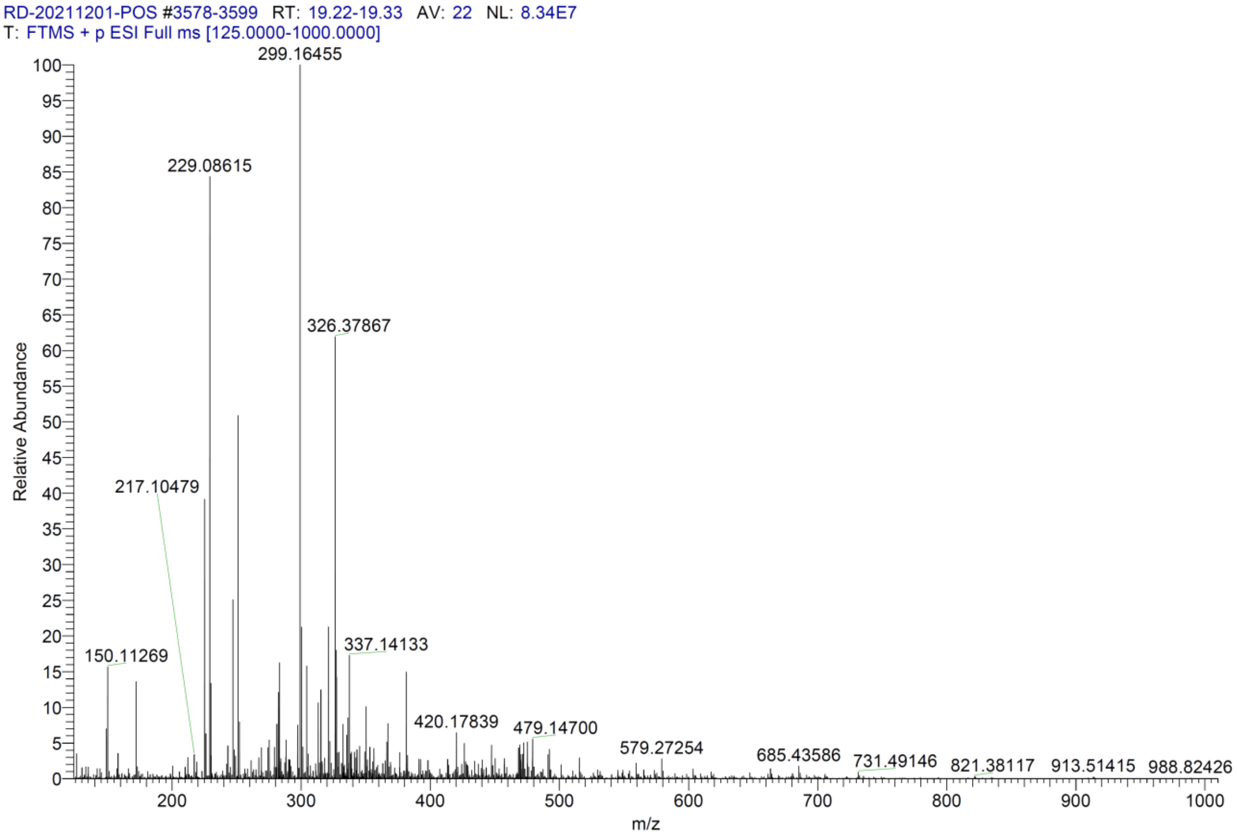

S21. Mass spectrum of **3**, positive mod

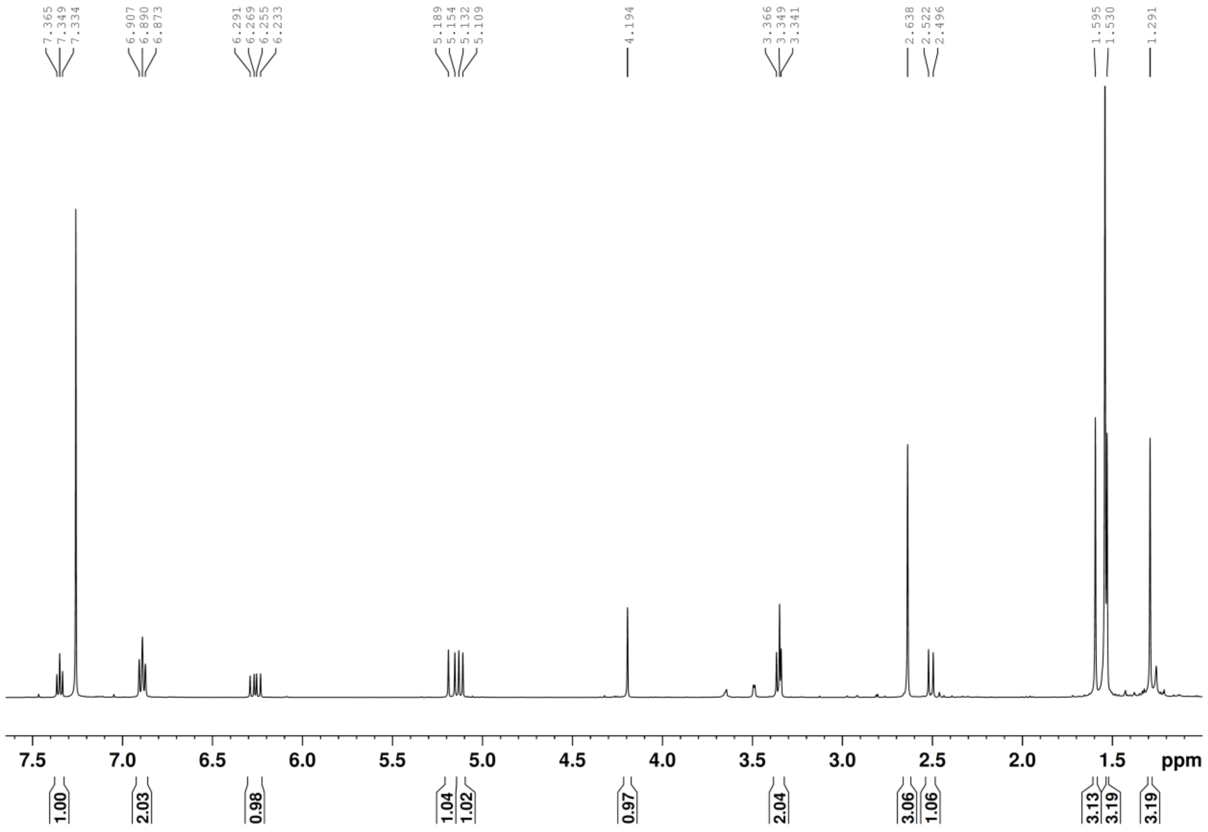

S22. ^{1}H NMR spectrum of **4**, $CDCl_3$, 298 K

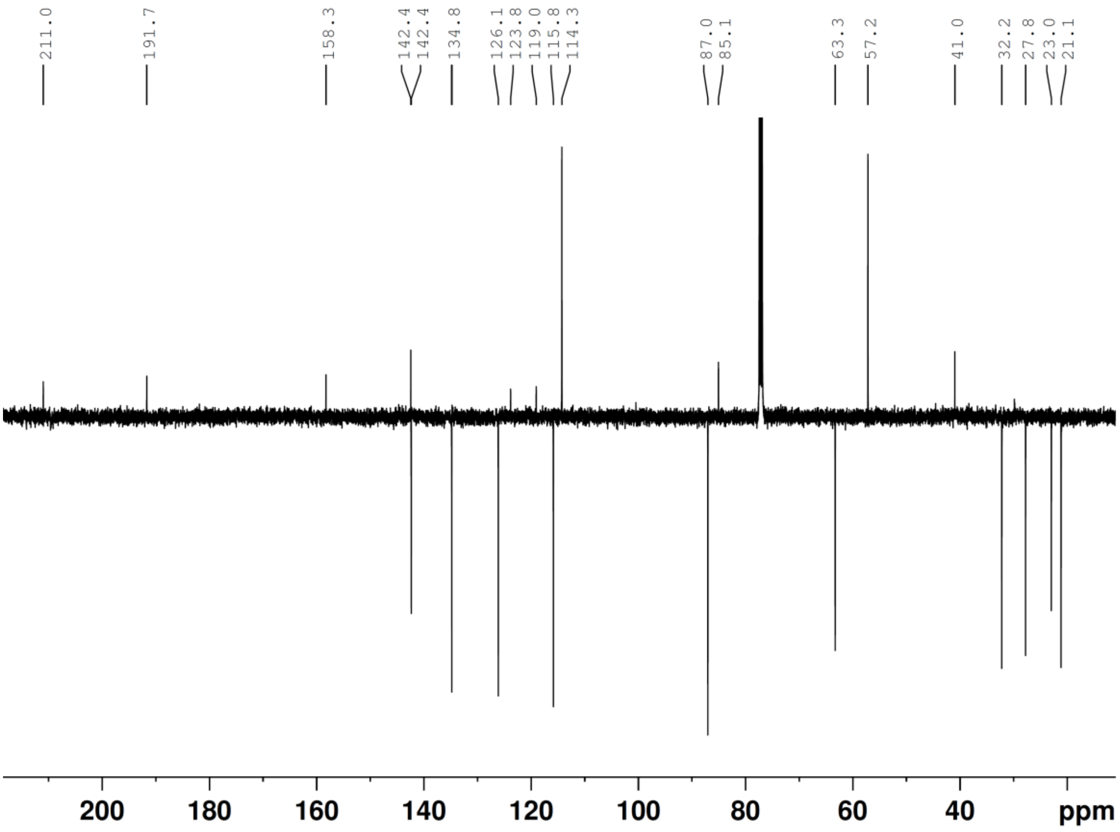

S23. ^{13}C NMR spectrum of **4**, $CDCl_3$, 298 K

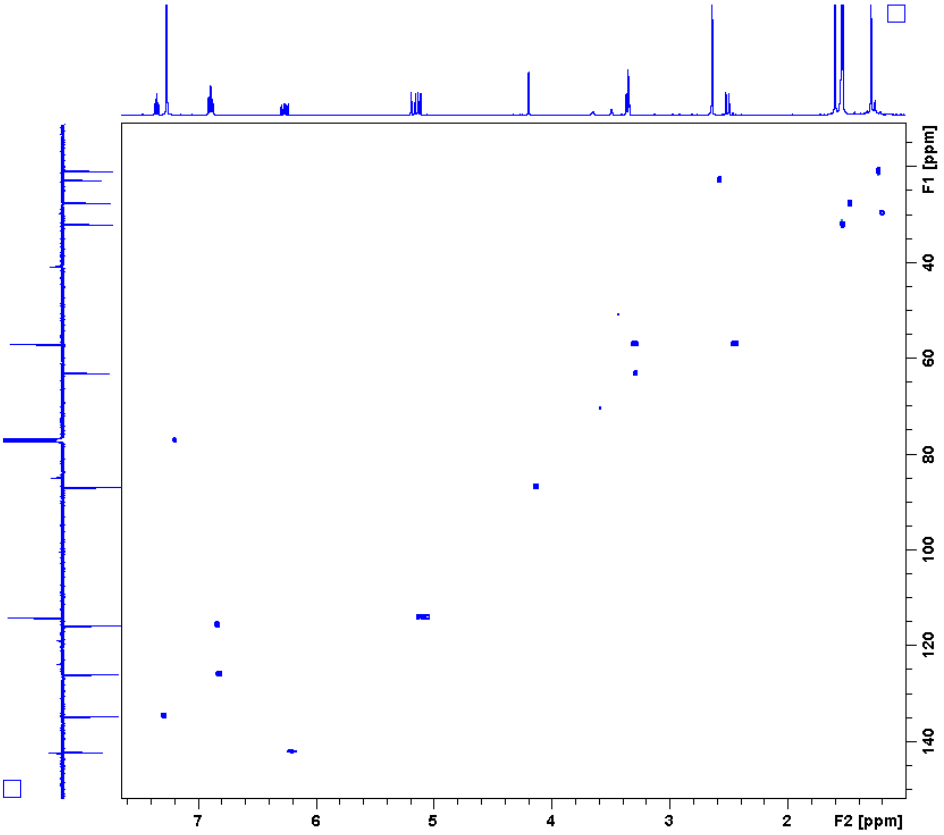

S24. HSQC spectrum of **4**, $CDCl_3$, 298 K

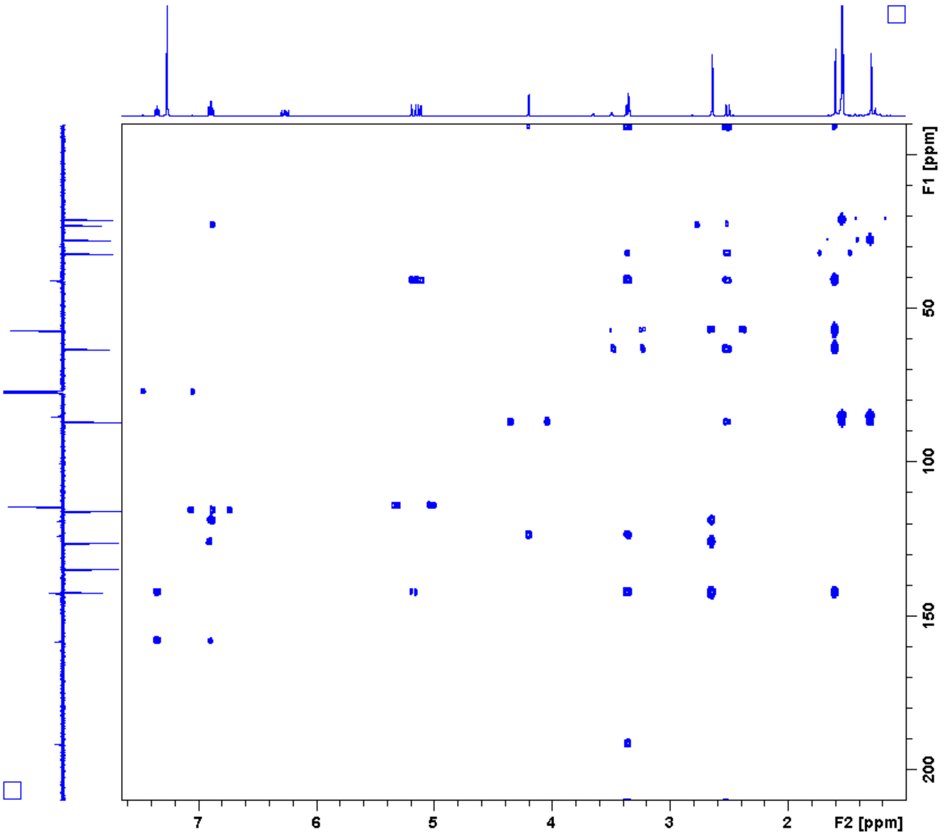

S25. HMBC spectrum of **4**, $CDCl_3$, 298 K

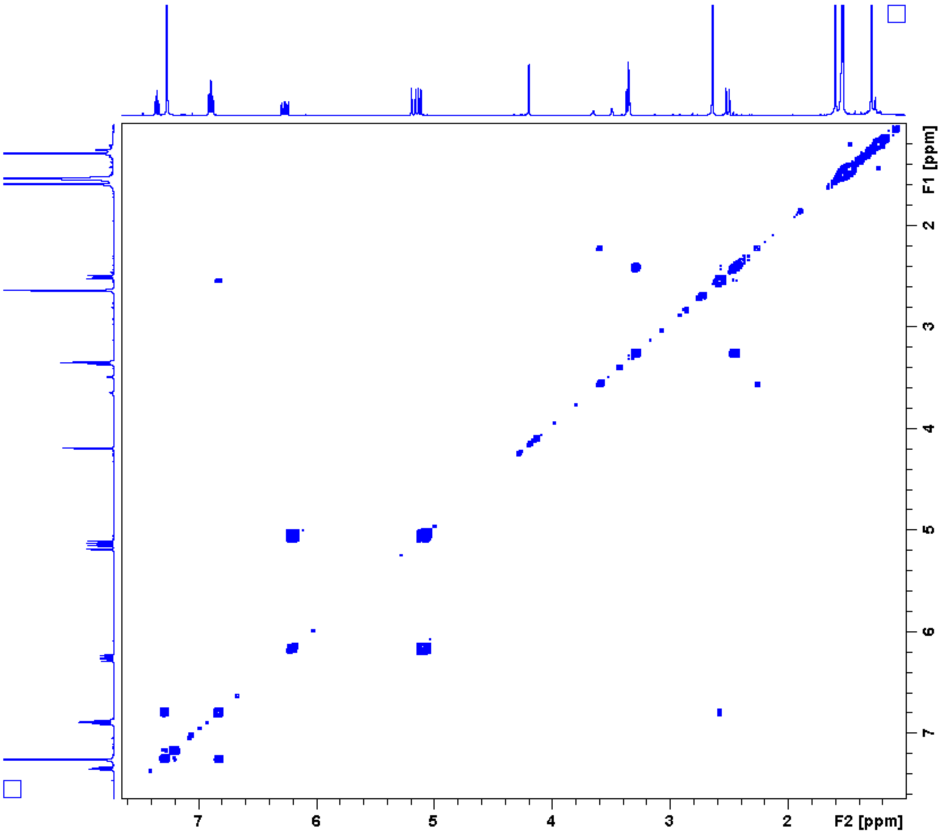

S26. COSY spectrum of **4**, $CDCl_3$, 298 K

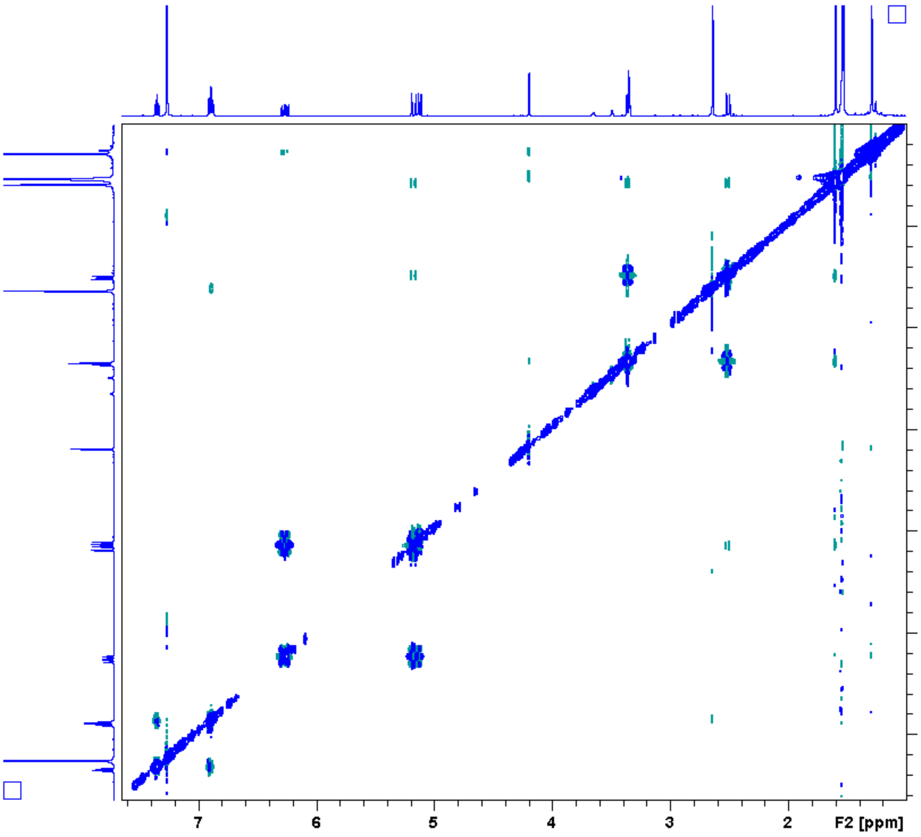

S27. NOESY spectrum of **4**, $CDCl_3$, 298 K

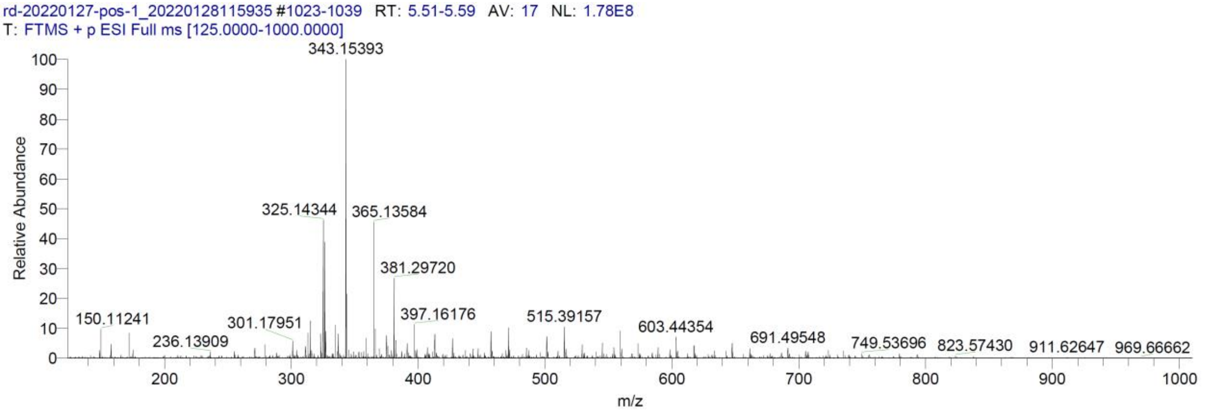

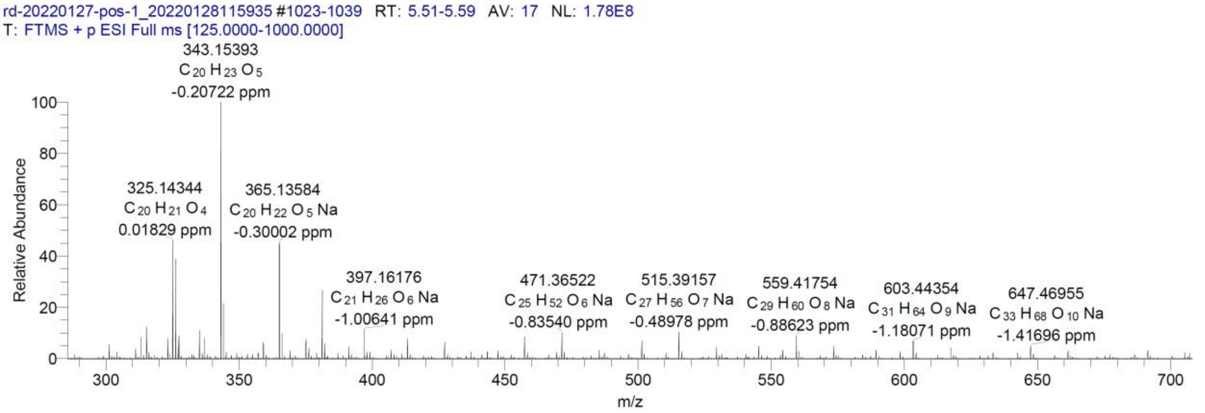

S28. Mass spectrum of **4**, positive mode

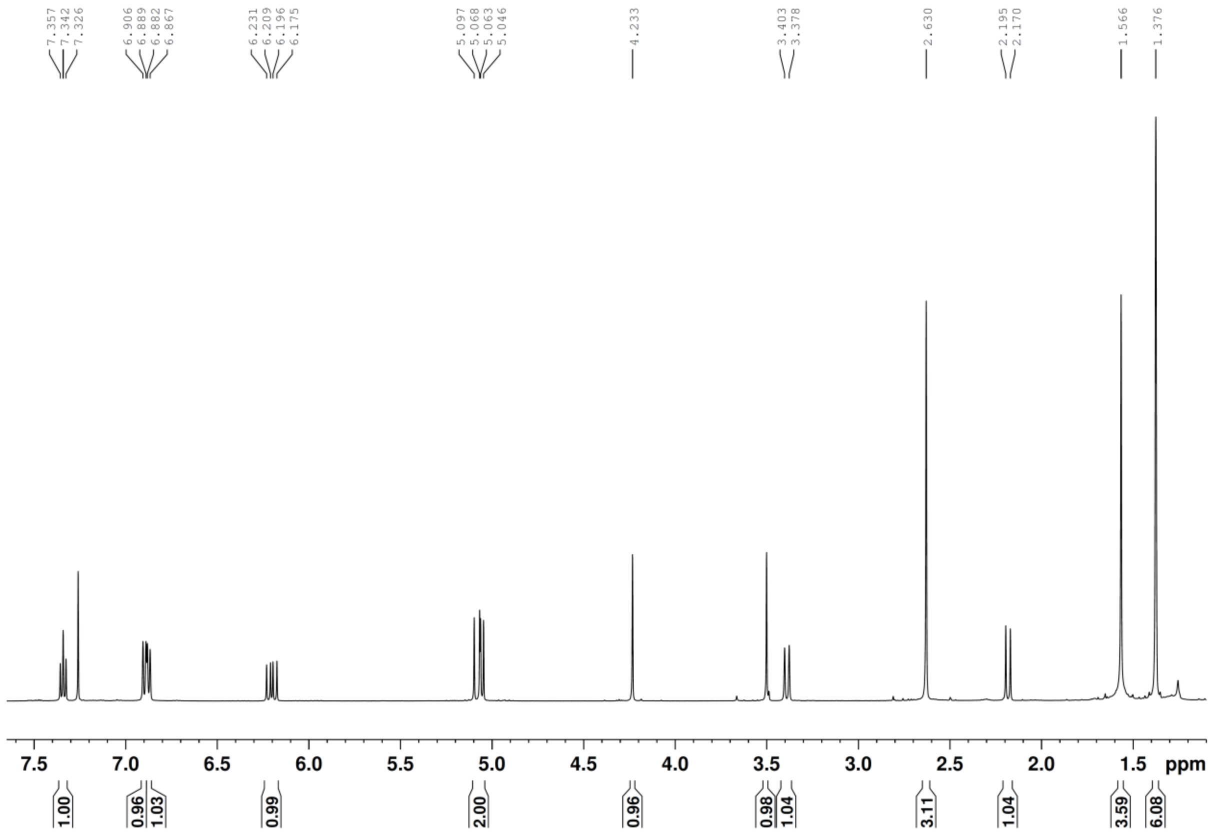

S29. ^{1}H NMR spectrum of **5**, $CDCl_3$, 298 K

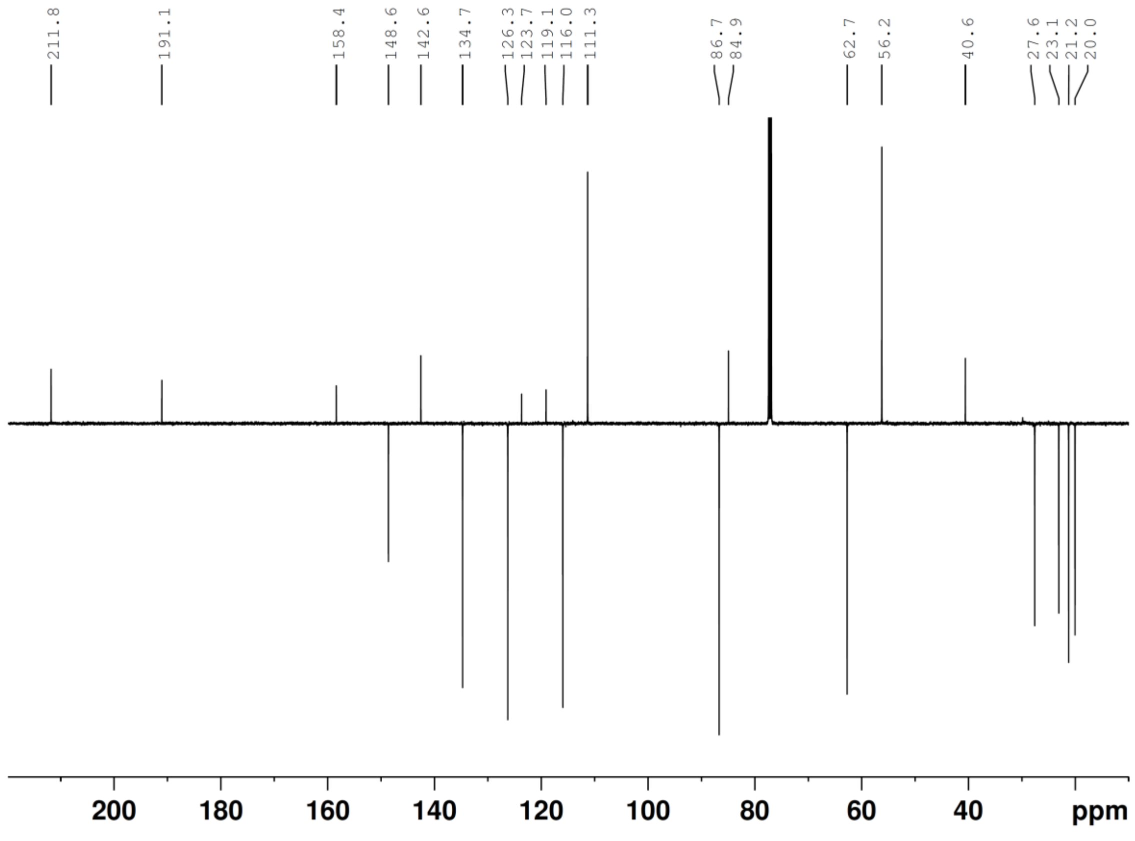

S30. ^{13}C NMR spectrum of **5**, $CDCl_3$, 298 K

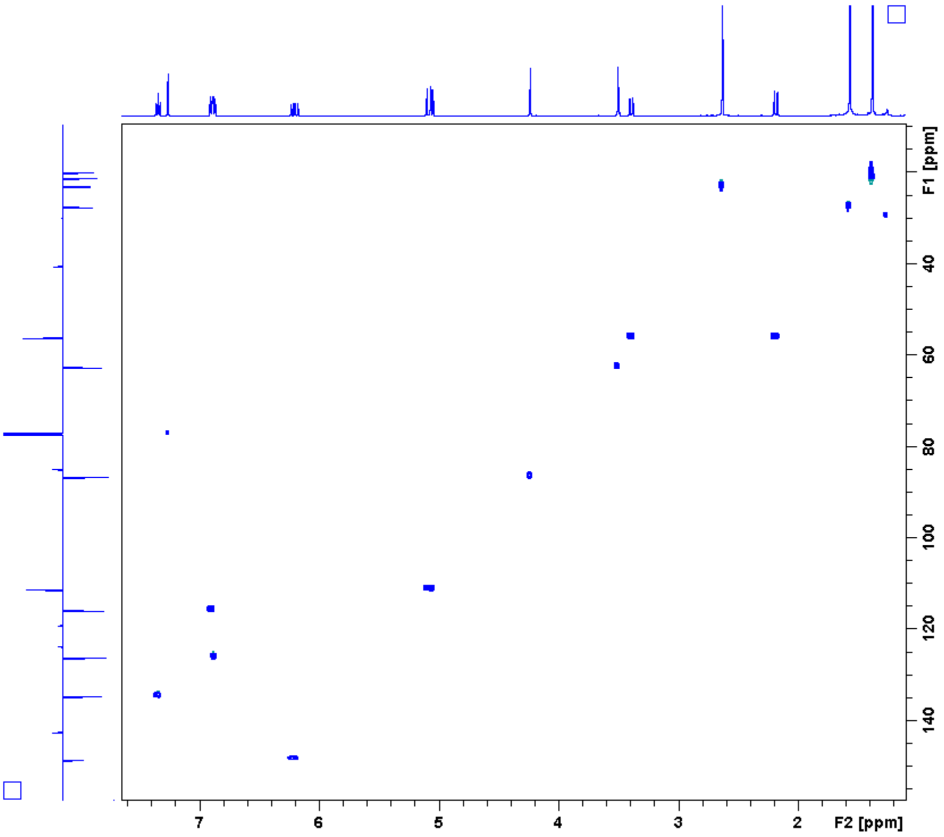

S31. HSQC spectrum of **5**, $CDCl_3$, 298 K

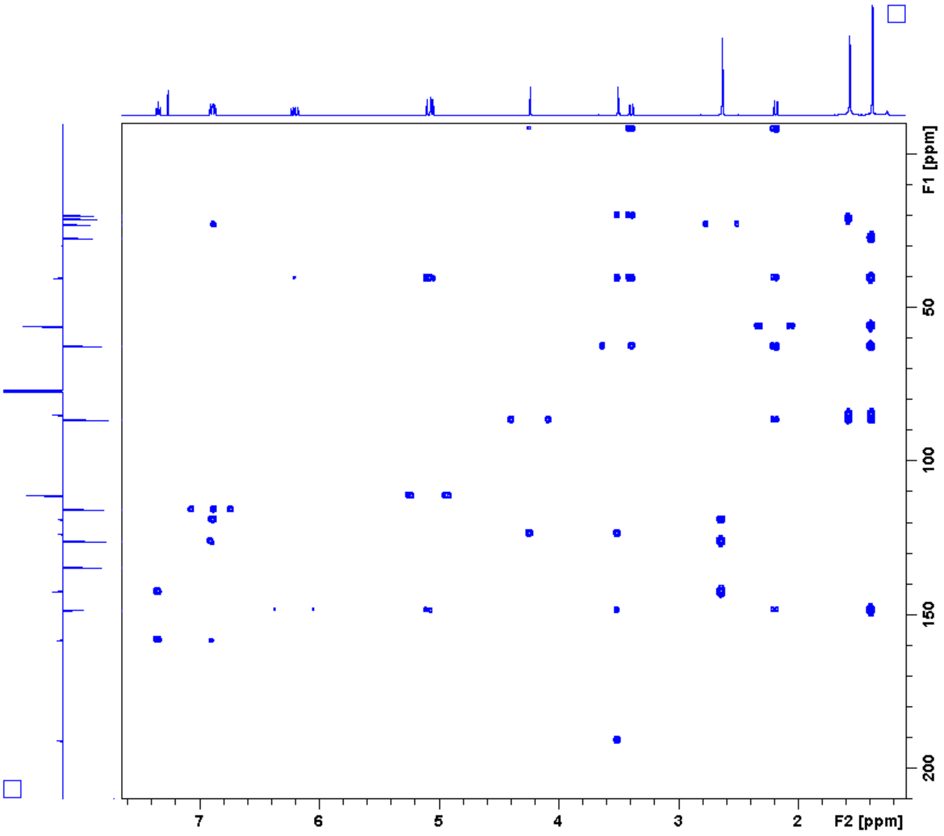

S32. HMBC spectrum of **5**, $CDCl_3$, 298 K

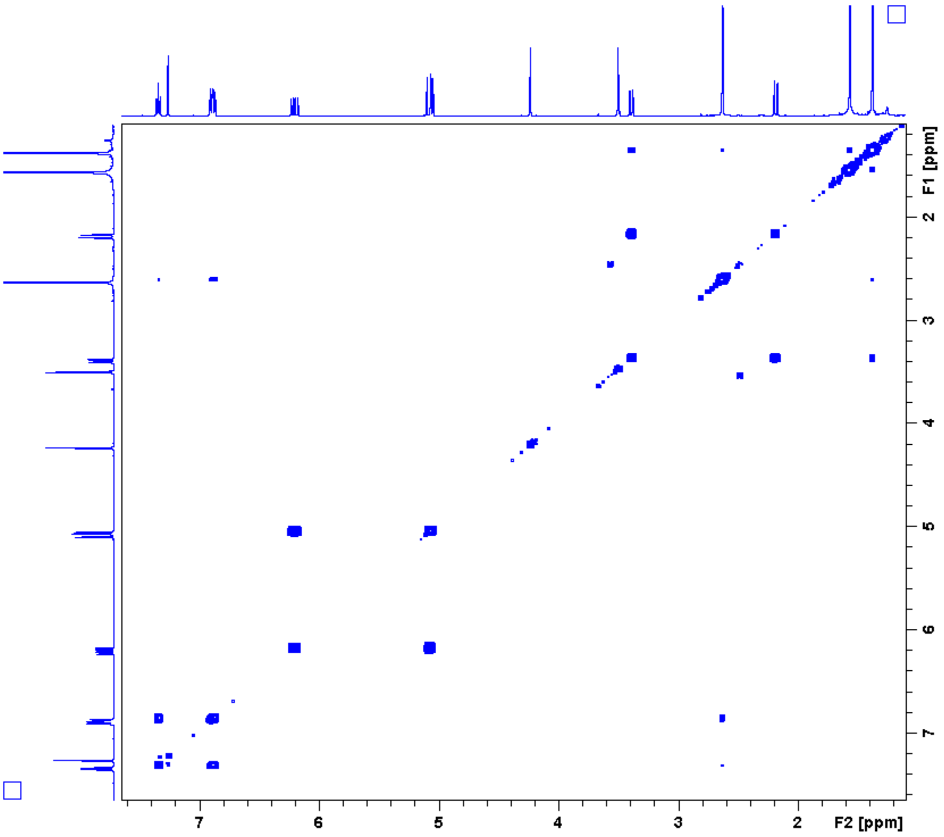

S33. COSY spectrum of **5**, $CDCl_3$, 298 K

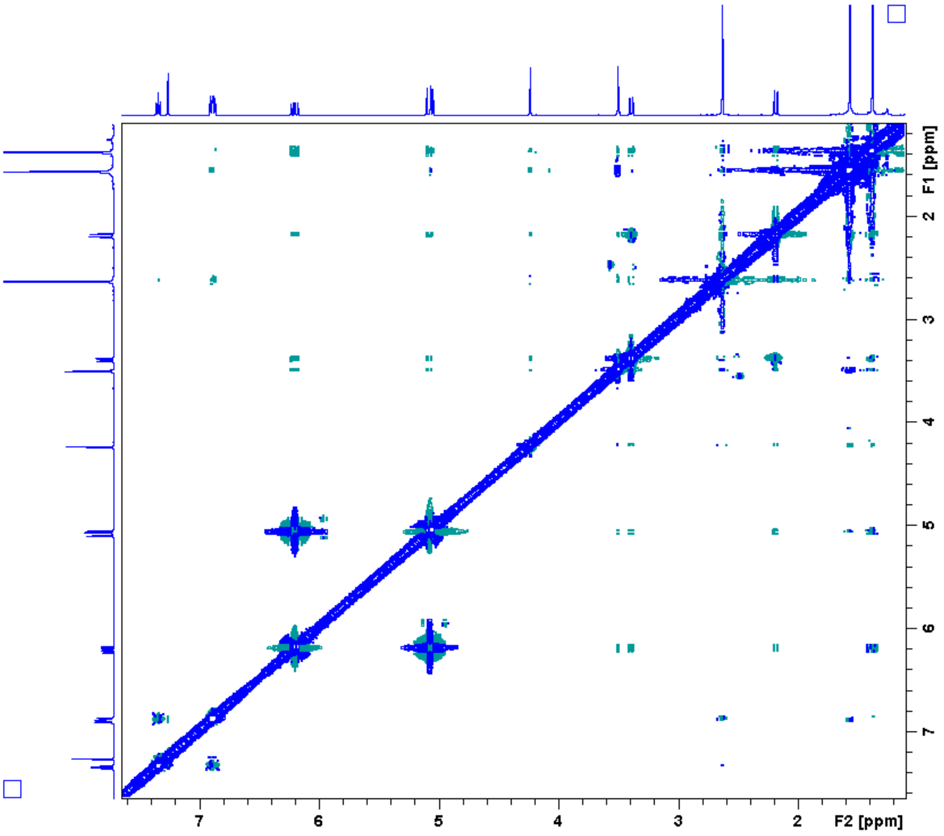

S34. NOESY spectrum of **5**, $CDCl_3$, 298 K

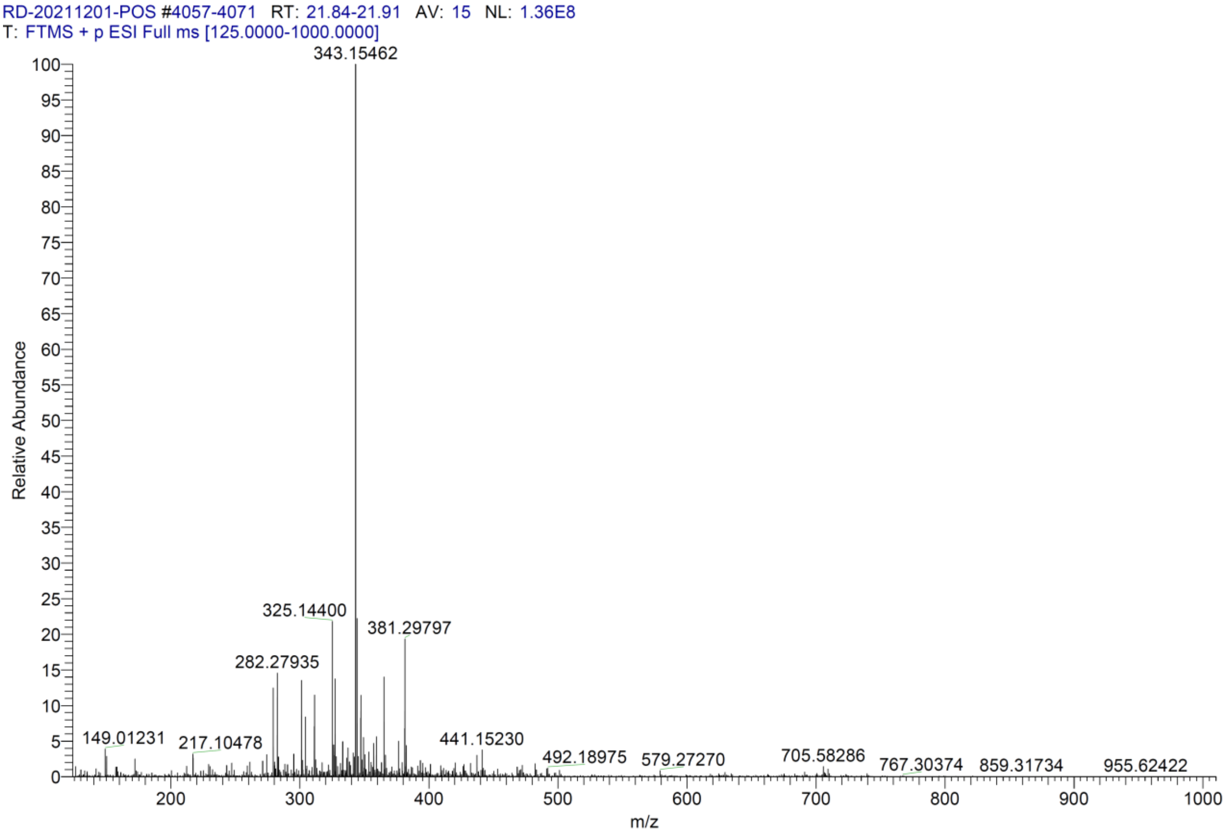

S35. Mass spectrum of **5**, $CDCl_3$, 298 K

*3. Structure determination of compound **1** by single crystal X-ray diffraction*

The well developed colourless, whitish transparent chunk type single crystals of **1** were grown from a mixture of methanol and ethyl acetate in two days at -5°C. The measured single crystal with the size of 0.5 x 0.4 x 0.4 mm was selected under the optical microscope and attached to the loop of the goniometerhead by parathion oil. The compound is chiral, light sensitive and thermally stable below -5°C, therefore the single crystal diffraction experiment was performed in dark at 103(2)K, using CuKα radiation. The atomic positions were determined by direct methods, all hydrogen atoms were found on the difference Fourier maps [2]. Crystal data and details of the structure determination and refinement are listed in Tables S2, atomic coordinates and equivalent isotropic displacement parameters are in Table S3, hydrogen coordinates and equivalent isotropic displacement parameters are in Table S4, anisotropic displacement parameters are listed in Table S5, bond length and angles, respectively can be found in Table S6 and S7, while intra- and intermolecular interactions of **1** are presented in Table S8.

1 crystallizes in the trigonal crystal system, in the chiral space group $P3_121$ (#152). There is one molecule in the asymmetric unit (Figure 1) Z=6, Z'=1. There is no residual solvent accessible void in the crystal lattice, which would be large enough to enclathrate small molecules. The Kitaigorodskii packing coefficient is 66.6% [3].

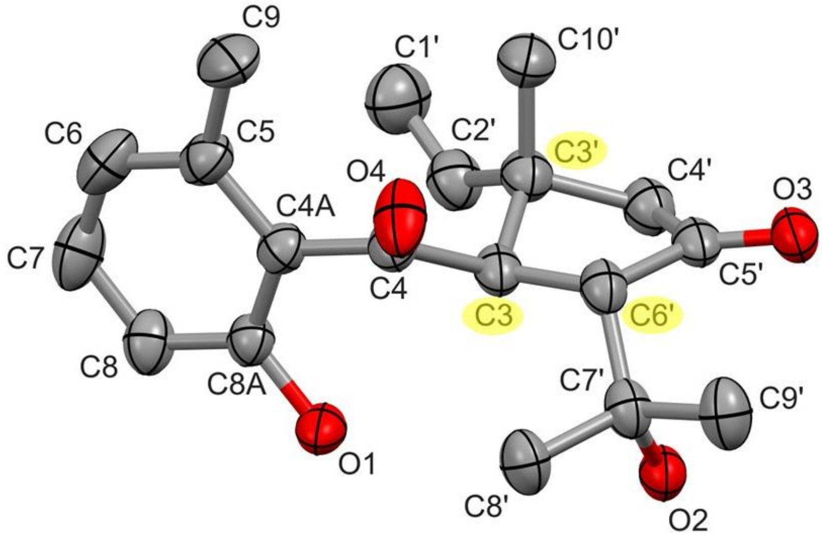

a.,

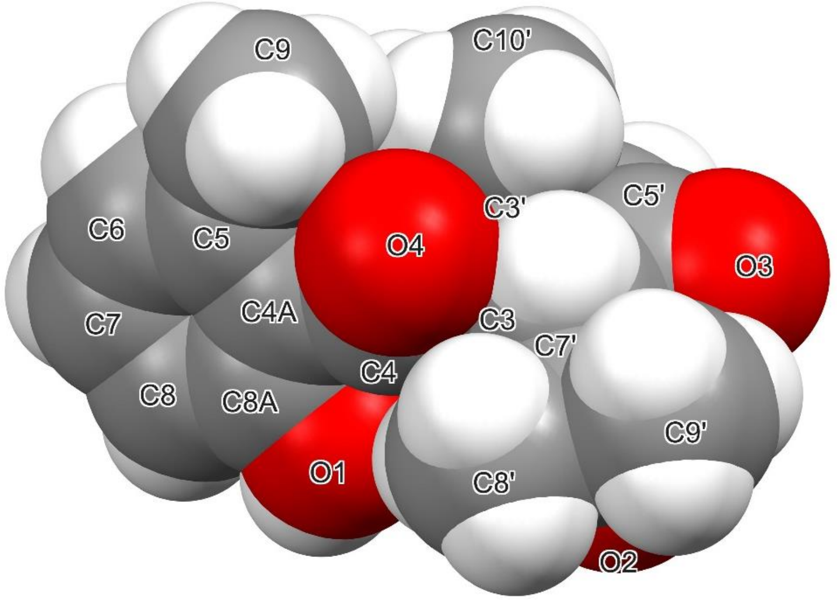

b.,

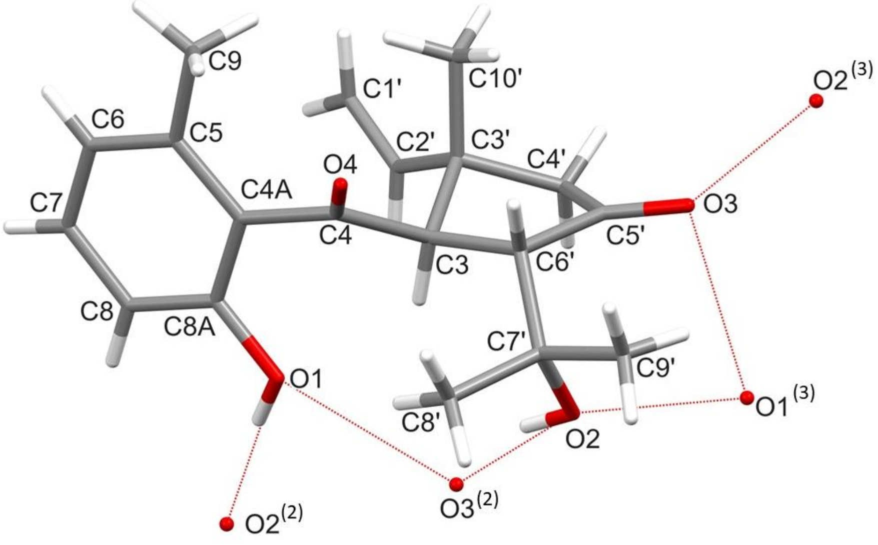

c.,

Figure S36 a., Molecular structure ORTEP presentation of compound **1** showing atom labelling. The displacement ellipsoids are drawn at the 50% probability level. The chiral centres are highlighted by yellow, C3 (R), C3’ (S) and C6’ (S). b., space filling model of **1**. c., Caped stick presentation showing the hydrogen atoms with the indication of the determining hydrogen bonds: O1-H1…O2 (molecule 2), O2-H2…O3 (molecule 2), O2…H1-O1 (molecule 3), O3…H2-O2 (molecule 3).

The absolute configuration has been established in the diffraction measurement (CuKα radiation) based on the anomalous-dispersion effect of the four oxygen atoms present in the molecule. The absolute configuration of the chiral atoms are C3 (R), C3’ (S) and C6’ (S). The Flack x= 0.11(5) [4], Parsons z = 0.09(5). Bijvoet pairs are 1293 (expected 1295), Friedel pair coverage 100%. A detailed analysis of the Bijvoet (Friedel) pairs found in an Fo/Fc reflection was performed to establish the absolute structure in terms of the Hooft parameter [5]. Bayesian statistics: P2(true) 1.000, Hooft y= 0.11(5).

The five membered ring has envelope conformation having C3' out of the plane with 0.634 Å (Figure S37a). C4 is in equatorial position on C3; C3' has two substituents: C2' in equatorial, while C10' in axial position; O3 can be found in equatorial position on C5'; and C7' is in bisectional position. The plane determined by C3, C4', C5' and C6', and the benzene ring of the molecule have the angle of 87.12°. O1 and O3 oxygen atoms participates in both intra- and intermolecular interactions. O2 forms only intermolecular interaction, while O4 is ill placed and forms only intramolecular interactions because of sterical hindrance. The molecular conformation is stabilized by four week C-H...O type hydrogen bonds (Figure 37b, Table S8).

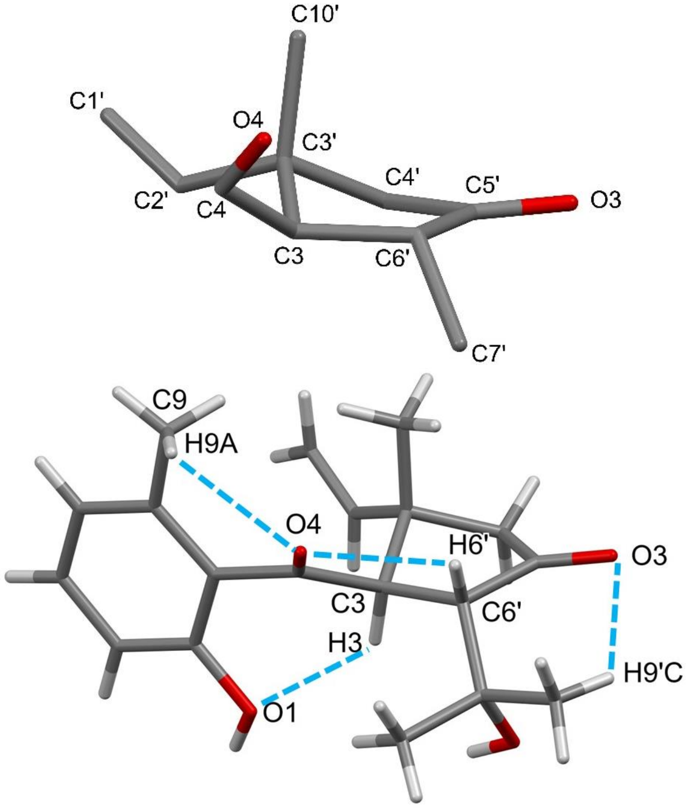

a., b.,

Figure S37 a., The conformation of the five membered ring and the ring substituents. b., Intramolecular interaction in **1** stabilizing the molecular conformation.

One molecule forms strong O-H...O type hydrogen bonds with two other molecules (Figure S36c, Table S8) by the O1-H1...O2 and O2-H2...O3 interactions, while O1 and O3 get close proximity of 3.027(2) Å [1+x-y,1-y,5/3-z]. These interactions connect the molecules into infinite chains. The chains are arranged along the 3_1 axes, which run parallel to the c crystallographic axis. Packing diagrams are presented in Figure S38. The diameter of the channel formed along the c crystallographic axis, which coincides with the 3_1 screw axis as symmetry element, is around 3.3 Å. The channel is hydrophobic, aromatic C-H and CH_3 groups form its wall. The phenyl rings in the crystal lattice can not get close to each other to form $\pi...\pi$ interactions, having the closest distance of 5.6016(16) Å [1-x,-x+y,4/3-z].

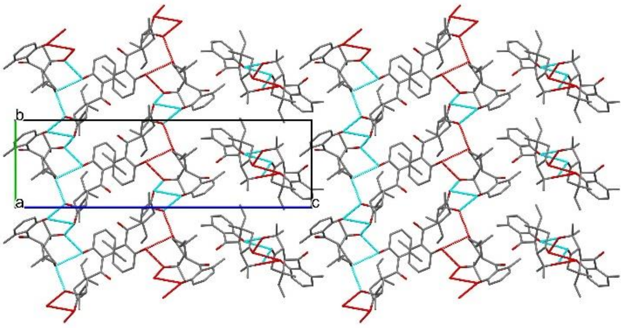

a.,

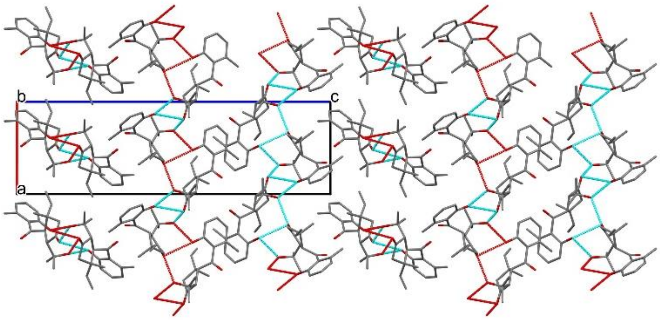

b.,

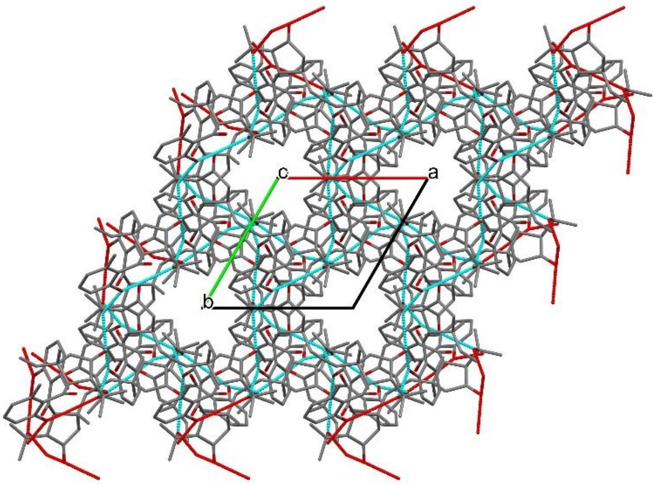

c.,

Figure S38 Packing diagram of **1** viewed along the a, b and c crystallographic axes, respectively.

The calculated morphology of the crystal of **1** is presented in Figure S39 assuming that the growing rates of all crystal sheets are equal. It agrees with the crystal habit received from the single crystal growth experiment.

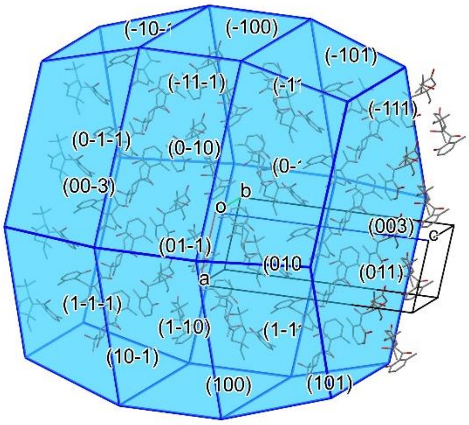

Figure S39 The calculated morphology of the crystal of **1**.

The powder pattern of **1** is calculated based on the single crystal structure with CuKα radiation in the 5 – 40° 2θ range (Figure S40).

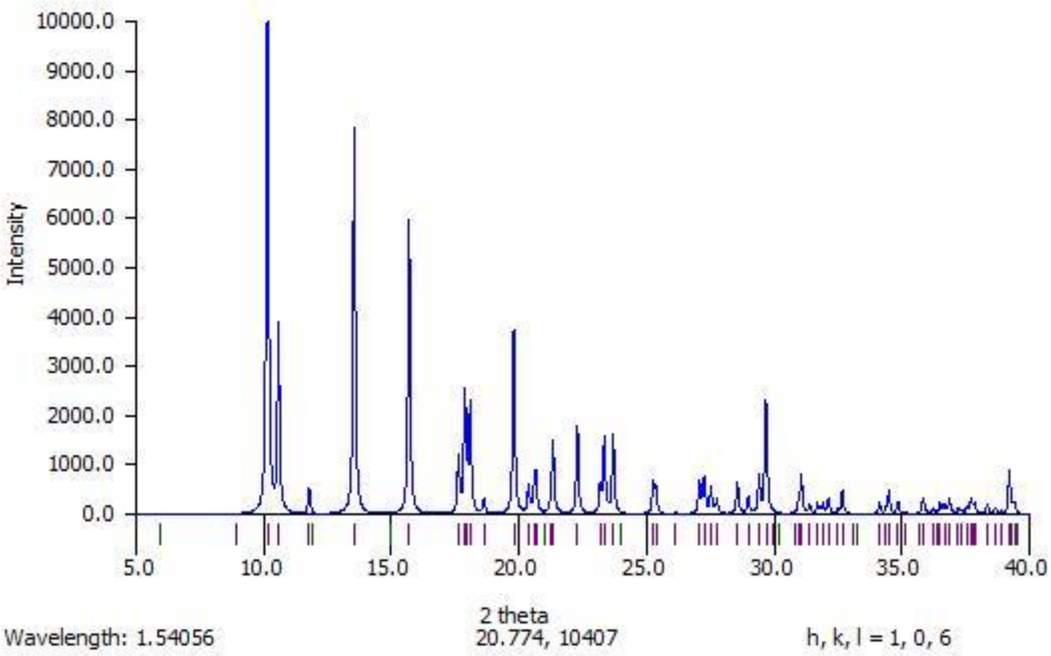

Figure S40 The calculated powder diffraction pattern of **1**.

Experimental

Crystal data of **1***:* $C_{19}H_{24}O_4$, *Fwt.*: 316.38, colourless, chunk, size: 0.5 x 0.4 x 0.4 mm, trigonal, space group *P* 3_1 2 1 (#152), a = 10.0520(3)Å, b = 10.0520(3)Å, c = 29.6147(9)Å, α = 90°, β = 90°, γ = 120°, V = 2591.45(17)Å^3, T = 103(2)K, Z= 6, Z'=1, F(000) = 1020, D_x = 1.216 Mg/m^3, μ 0.681mm^{-1}.
A crystal of **1** was mounted on a loop. Cell parameters were determined by least-squares using

62994 (4.479° ≤ θ ≤ 68.251°) reflections. Intensity data were collected on an RAXIS-RAPID II diffractometer (graphite monochromator; Cu-*K*α radiation, λ = 1.54178Å) at 103(2) K in the range 4.479° ≤ θ ≤ 68.251° [6]. A total of 62994 reflections were collected of which 3155 were unique [*R*(int) = 0.0773, *R*(σ) = 0.0238]; intensities of 3088 reflections were greater than 2σ(*I*). Completeness to θ = 0.999.

A numerical absorption correction was applied to the data (the minimum and maximum transmission factors were 0.94800 and 0.95530). [7]

The structure was solved by direct methods [2]

Anisotropic full-matrix least-squares refinement [2k] on F^2 for all non-hydrogen atoms yielded *R*1 = 0.0344 and *wR*2 = 0.0865 for 1332 [*I*>2σ(*I*)] and *R*1 = 0.0337 and *wR*2 = 0.0871 for all 3155) intensity data, (number of parameters = 222, goodness-of-fit = 1.069, the maximum and mean shift/esd is 0.001 and 0.000). The absolute structure parameter is 0.11(5). (Friedel coverage: 1.000).

The maximum and minimum residual electron density in the final difference map was 0.17 and -0.13e.$Å^{-3}$.

The weighting scheme applied was $w = 1/[\sigma^2(F_o^2)+(0.0455P)^2+0.3383P]$, where $P = (F_o^2+2F_c^2)/3$.

Hydrogen atomic positions were located in difference maps. Hydrogen atoms were included in structure factor calculations but they were not refined. The isotropic displacement parameters of the hydrogen atoms were approximated from the *U*(eq) value of the atom they were bonded to.

CCDC-2224099 (compound **1**) contains the supplementary crystallographic data for this paper. These data can be obtained free of charge from The Cambridge Crystallographic Data Centre via www.ccdc.cam.ac.uk/data_request/cif.

Table S2 Crystal data and details of the structure determination and refinement of **1**

Crystal Data			
Formula			$C_{19}H_{24}O_4$
Formula Weight			316.38
Crystal System			trigonal
Space group		$P3_12_1$	(No.152)
a, b, c [Å]	10.0520(3)	10.0520(3)	29.6147(9)
alpha, beta, gamma [°]	90	90	120
V [$Å^3$]			2591.45(17)
Z, Z'			6, 4
D(calc) [g/cm^3]			1.216
Mu(CuKα) [1/mm]			0.681
F(000)			1020
Crystal Size [mm]			0.4 x 0.4 x 0.5
**Data Collection**			
Temperature (K)			103
Radiation [Å]		CuKα	1.54187
Theta Min-Max [°]			4.5, 68.3
Dataset			-12: 11 ; -12: 12 ; -35: 35
Tot., Uniq. Data, R(int)			62994, 3155, 0.077
Observed Data [I > 2.0 σ(I)]			3088
**Refinement**			
Nref, Npar			3155, 222
R, wR2, S			0.0337, 0.0871, 1.07
$w = 1/[\sigma^2(F_o^2)+(0.0455P)^2+0.3383P]$, where $P = (F_o^2+2F_c^2)/3$			
Max. and Av. Shift/Error			0.00, 0.00
Flack x			0.11(5)
Min. and Max. Resd. Dens. [e/$Å^3$]			-0.13, 0.17

Table S3 Atomic coordinates and equivalent isotropic displacement parameters of **1**. *U*(eq) is defined as one third of the trace of the orthogonalized U_{ij} tensor.

Atom	x	y	z	U(eq) [Å²]
O1	0.47636(16)	0.60821(18)	0.77253(4)	0.0459(4)
O2	0.03943(16)	0.35290(17)	0.83220(4)	0.0432(4)
O3	-0.18898(17)	0.48032(19)	0.80149(4)	0.0469(4)
O4	0.17891(18)	0.5186(2)	0.68043(5)	0.0651(6)
C1'	0.4081(3)	1.0230(4)	0.75917(11)	0.0710(10)
C2'	0.3343(3)	0.8868(3)	0.77717(8)	0.0525(7)
C3	0.1861(2)	0.6030(2)	0.75624(6)	0.0408(6)
C3'	0.1813(2)	0.7551(3)	0.76314(7)	0.0445(6)
C4	0.2585(2)	0.5985(3)	0.71110(6)	0.0434(6)
C4'	0.0640(2)	0.7046(3)	0.80197(7)	0.0476(7)
C4A	0.4279(2)	0.7023(2)	0.70492(6)	0.0397(6)
C5	0.4845(3)	0.7962(3)	0.66630(7)	0.0468(6)
C5'	-0.0544(2)	0.5432(3)	0.79047(6)	0.0416(6)
C6	0.6410(3)	0.8973(3)	0.66298(7)	0.0580(7)
C6'	0.0199(2)	0.4709(3)	0.76243(6)	0.0417(6)
C7	0.7423(3)	0.9029(3)	0.69534(8)	0.0615(8)
C7'	0.0002(2)	0.3231(2)	0.78494(7)	0.0433(6)
C8	0.6896(2)	0.8067(3)	0.73230(7)	0.0494(7)
C8'	0.1020(3)	0.2701(3)	0.76256(7)	0.0522(7)
C8A	0.5326(2)	0.7078(2)	0.73716(6)	0.0393(6)
C9	0.3824(3)	0.7905(4)	0.62813(8)	0.0669(10)
C9'	-0.1665(3)	0.1948(3)	0.78415(8)	0.0565(8)
C10'	0.1137(3)	0.7926(3)	0.72200(7)	0.0549(8)

U(eq) = 1/3 of the trace of the orthogonalized U Tensor

Table S4 Hydrogen coordinates and isotropic displacement parameters of **1**.

Atom	x	y	z	U(iso) [Å^2]
H1	0.54557	0.62832	0.79066	0.0690
H1'A	0.512(4)	1.106(4)	0.7702(10)	0.079(9)
H2	0.13288	0.40630	0.83485	0.0650
H1'B	0.372(4)	1.048(4)	0.7329(11)	0.089(10)
H3	0.24851	0.59591	0.78053	0.0490
H2'A	0.38180	0.86897	0.80151	0.0630
H4'A	0.11238	0.70863	0.83068	0.0570
H4'B	0.01854	0.76978	0.80358	0.0570
H6	0.67870	0.96309	0.63830	0.0700
H6'	-0.02993	0.44383	0.73277	0.0500
H8'A	0.20789	0.34754	0.76624	0.0780
H7	0.84681	0.97197	0.69223	0.0740
H8'B	0.07800	0.25305	0.73097	0.0780
H8	0.75808	0.80796	0.75365	0.0590
H8'C	0.08450	0.17622	0.77635	0.0780
H9'A	-0.17392	0.10278	0.79632	0.0850
H9A	0.33037	0.68907	0.61543	0.1010
H9B	0.44384	0.86316	0.60527	0.1010
H9C	0.30819	0.81570	0.63940	0.1010
H9'B	-0.20340	0.17656	0.75361	0.0850
H10A	0.18439	0.82213	0.69721	0.0820
H10B	0.09590	0.87548	0.72927	0.0820
H10C	0.01828	0.70360	0.71373	0.0820
H9'C	-0.22742	0.22399	0.80203	0.0850

Table S5 Anisotropic displacement parameters of **1**. The anisotropic displacement factor exponent takes the form: $-2\pi^2(h^2a^{*2}U_{11} + ... + 2hka^*b^*U_{12})$.

Atom	U(1,1) or U	U(2,2)	U(3,3)	U(2,3)	U(1,3)	U(1,2)
----	------	------	------	------	------	------
O1	0.0369(7)	0.0618(9)	0.0370(7)	0.0101(6)	-0.0008(6)	0.0232(7)
O2	0.0373(7)	0.0548(9)	0.0387(7)	0.0021(6)	0.0048(6)	0.0239(7)
O3	0.0393(7)	0.0666(9)	0.0410(7)	0.0037(7)	0.0038(6)	0.0311(7)
O4	0.0428(8)	0.1033(14)	0.0353(7)	-0.0105(8)	-0.0027(6)	0.0262(9)
C1'	0.0524(15)	0.0647(17)	0.0808(19)	0.0060(14)	-0.0024(14)	0.0179(14)
C2'	0.0450(12)	0.0594(14)	0.0515(11)	-0.0035(10)	-0.0033(9)	0.0249(11)
C3	0.0353(10)	0.0571(12)	0.0317(9)	0.0015(8)	-0.0006(7)	0.0244(10)
C3'	0.0409(11)	0.0554(13)	0.0389(9)	0.0012(9)	-0.0010(8)	0.0253(10)
C4	0.0391(10)	0.0618(13)	0.0311(9)	0.0029(9)	0.0000(8)	0.0266(10)
C4'	0.0464(12)	0.0586(13)	0.0427(10)	-0.0030(9)	0.0010(9)	0.0300(11)
C4A	0.0411(10)	0.0456(11)	0.0329(8)	0.0010(8)	0.0026(8)	0.0221(9)
C5	0.0571(12)	0.0494(12)	0.0364(9)	0.0059(9)	0.0084(9)	0.0284(10)
C5'	0.0402(11)	0.0610(13)	0.0307(8)	0.0032(8)	-0.0010(8)	0.0306(10)
C6	0.0679(15)	0.0450(12)	0.0457(11)	0.0084(10)	0.0138(11)	0.0166(12)
C6'	0.0348(10)	0.0570(13)	0.0335(9)	-0.0029(8)	0.0000(8)	0.0230(10)
C7	0.0469(13)	0.0522(14)	0.0571(13)	-0.0040(11)	0.0112(11)	0.0035(11)
C7'	0.0369(11)	0.0526(12)	0.0400(10)	-0.0068(8)	0.0016(8)	0.0221(9)
C8	0.0385(11)	0.0533(13)	0.0459(10)	-0.0062(9)	-0.0005(9)	0.0150(10)
C8'	0.0477(12)	0.0582(13)	0.0549(12)	-0.0063(10)	0.0075(10)	0.0296(11)
C8A	0.0389(10)	0.0443(11)	0.0339(8)	0.0004(8)	0.0040(7)	0.0201(9)
C9	0.0808(18)	0.094(2)	0.0409(11)	0.0210(12)	0.0094(12)	0.0550(16)
C9'	0.0423(12)	0.0564(14)	0.0625(13)	-0.0119(11)	0.0041(10)	0.0184(11)
C10'	0.0494(13)	0.0692(15)	0.0499(11)	0.0097(11)	-0.0010(10)	0.0325(12)

Table S6 Bond lengths [Å] of **1**.

O1	-C8A	1.361(2)	C7'	-C9'	1.520(4)
O2	-C7'	1.444(2)	C8	-C8A	1.390(3)
O3	-C5'	1.217(3)	C1'	-H1'A	1.01(4)
O4	-C4	1.211(3)	C1'	-H1'B	0.94(4)
O1	-H1	0.8200	C2'	-H2'A	0.9300
O2	-H2	0.8200	C3	-H3	0.9800
C1'	-C2'	1.301(4)	C4'	-H4'A	0.9700
C2'	-C3'	1.502(4)	C4'	-H4'B	0.9700
C3	-C6'	1.539(3)	C6	-H6	0.9300
C3	-C3'	1.567(3)	C6'	-H6'	0.9800
C3	-C4	1.534(3)	C7	-H7	0.9300
C3'	-C10'	1.531(3)	C8	-H8	0.9300
C3'	-C4'	1.540(3)	C8'	-H8'A	0.9600
C4	-C4A	1.498(3)	C8'	-H8'B	0.9600
C4'	-C5'	1.495(4)	C8'	-H8'C	0.9600
C4A	-C5	1.409(3)	C9	-H9A	0.9600
C4A	-C8A	1.402(3)	C9	-H9B	0.9600
C5	-C9	1.509(4)	C9	-H9C	0.9600
C5	-C6	1.385(4)	C9'	-H9'A	0.9600
C5'	-C6'	1.523(3)	C9'	-H9'B	0.9600
C6	-C7	1.379(4)	C9'	-H9'C	0.9600
C6'	-C7'	1.548(3)	C10'	-H10A	0.9600
C7	-C8	1.379(3)	C10'	-H10B	0.9600
C7'	-C8'	1.522(4)	C10'	-H10C	0.9600

Table S7 Bond angles [°] of **1**.

C8A	-O1	-H1	109.00	C3	-C6'	-C5'	103.48(19)
C7'	-O2	-H2	109.00	C6	-C7	-C8	120.4(3)
C1'	-C2'	-C3'	127.5(3)	O2	-C7'	-C9'	105.17(17)
C3'	-C3	-C6'	106.05(19)	O2	-C7'	-C6'	109.12(15)
C4	-C3	-C6'	113.88(16)	O2	-C7'	-C8'	109.45(18)
C3'	-C3	-C4	112.90(17)	C8'	-C7'	-C9'	110.20(19)
C2'	-C3'	-C10'	112.8(2)	C6'	-C7'	-C8'	111.27(18)
C2'	-C3'	-C3	112.3(2)	C6'	-C7'	-C9'	111.4(2)
C2'	-C3'	-C4'	111.22(19)	C7	-C8	-C8A	119.0(2)
C4'	-C3'	-C10'	108.3(2)	C4A	-C8A	-C8	121.30(17)
C3	-C3'	-C10'	111.74(18)	O1	-C8A	-C4A	117.88(18)
C3	-C3'	-C4'	99.66(18)	O1	-C8A	-C8	120.75(19)
O4	-C4	-C4A	120.93(19)	C2'	-C1'	-H1'A	123.1(19)
C3	-C4	-C4A	118.48(17)	C2'	-C1'	-H1'B	122(2)
O4	-C4	-C3	120.5(2)	H1'A	-C1'	-H1'B	115(3)
C3'	-C4'	-C5'	104.51(18)	C1'	-C2'	-H2'A	116.00
C4	-C4A	-C8A	120.92(16)	C3'	-C2'	-H2'A	116.00
C4	-C4A	-C5	120.2(2)	C3'	-C3	-H3	108.00
C5	-C4A	-C8A	118.9(2)	C4	-C3	-H3	108.00
C4A	-C5	-C9	122.9(3)	C6'	-C3	-H3	108.00
C6	-C5	-C9	118.4(2)	C3'	-C4'	-H4'A	111.00
C4A	-C5	-C6	118.6(2)	C3'	-C4'	-H4'B	111.00
C4'	-C5'	-C6'	109.2(2)	C5'	-C4'	-H4'A	111.00
O3	-C5'	-C4'	125.3(2)	C5'	-C4'	-H4'B	111.00
O3	-C5'	-C6'	125.6(2)	H4'A	-C4'	-H4'B	109.00
C5	-C6	-C7	121.7(2)	C5	-C6	-H6	119.00
C5'	-C6'	-C7'	111.43(17)	C7	-C6	-H6	119.00
C3	-C6'	-C7'	116.23(19)	C3	-C6'	-H6'	108.00
C5'	-C6'	-H6'	108.00	H9A	-C9	-H9B	109.00
C7'	-C6'	-H6'	108.00	H9A	-C9	-H9C	110.00
C6	-C7	-H7	120.00	H9B	-C9	-H9C	109.00
C8	-C7	-H7	120.00	C7'	-C9'	-H9'A	109.00
C7	-C8	-H8	121.00	C7'	-C9'	-H9'B	109.00
C8A	-C8	-H8	120.00	C7'	-C9'	-H9'C	109.00
C7'	-C8'	-H8'A	109.00	H9'A	-C9'	-H9'B	109.00
C7'	-C8'	-H8'B	110.00	H9'A	-C9'	-H9'C	109.00
C7'	-C8'	-H8'C	110.00	H9'B	-C9'	-H9'C	109.00
H8'A	-C8'	-H8'B	109.00	C3'	-C10'	-H10A	109.00
H8'A	-C8'	-H8'C	109.00	C3'	-C10'	-H10B	109.00
H8'B	-C8'	-H8'C	109.00	C3'	-C10'	-H10C	109.00
C5	-C9	-H9A	109.00	H10A	-C10'	-H10B	109.00
C5	-C9	-H9B	109.00	H10A	-C10'	-H10C	109.00
C5	-C9	-H9C	109.00	H10B	-C10'	-H10C	109.00

Table S8 Intra- and intermolecular interactions in **1**.

D-H…A	D-H [Å]	H…A [Å]	D…A [Å]	D-H…A [°]	symmetry operation
O1-H1...O2	0.8200	1.8600	2.675(3)	172.00	1+x-y,1-y,5/3-z
O2-H2...O3	0.8200	1.9500	2.726(3)	158.00	1+x-y,1-y,5/3-z
C3-H3...O1	0.9800	2.2400	2.932(3)	126.00	intra
C6'-H6'...O4	0.9800	2.4100	2.813(3)	104.00	intra
C9-H9A...O4	0.9600	2.5200	2.909(4)	104.00	intra
C9'-H9'C...O3	0.9600	2.4100	3.033(3)	123.00	intra

4. Reference

1. Mosmann, T. J. *Immunol. Methods* (1983) 65, 55–63.

2. Sheldrick, G. M. *Acta Cryst*. (2008) A64, 112-122.

3. Spek, A. L. *J. Appl. Cryst*. (2003) 36, 7-13.

4. Flack, H. D. *Acta Cryst*. (1983) A39, 876-881.

5. Hooft, R. W. W.; Straver, L. H.; Spek, A. L. *J. Appl. Cryst.* (2008), 41, 96-103

6. CrystalClear SM 1.4.0 (Rigaku/MSC Inc., 2008).

7. Higashi, T. Numerical Absorption Correction, NUMABS, 2002
